# Association of Bundled Payments for Joint Replacement Surgery and Patient Outcomes With Simultaneous Hospital Participation in Accountable Care Organizations

**DOI:** 10.1001/jamanetworkopen.2019.12270

**Published:** 2019-09-27

**Authors:** Joshua M. Liao, Ezekiel J. Emanuel, Atheendar S. Venkataramani, Qian Huang, Claire T. Dinh, Eric Z. Shan, Erkuan Wang, Jingsan Zhu, Deborah S. Cousins, Amol S. Navathe

**Affiliations:** 1 Department of Medicine, University of Washington School of Medicine, Seattle; 2 Leonard Davis Institute of Health Economics, University of Pennsylvania, Philadelphia; 3 Department of Medical Ethics and Health Policy, Perelman School of Medicine, University of Pennsylvania, Philadelphia; 4 Corporal Michael J. Crescenz Veterans Affairs Medical Center, Philadelphia, Pennsylvania

## Abstract

**Importance:**

An increasing number of hospitals have participated in Medicare’s bundled payment and accountable care organization (ACO) programs. Although participation in bundled payments has been associated with savings for lower-extremity joint replacement (LEJR) surgery, simultaneous participation in ACOs may be associated with different outcomes given the prevalence of LEJR among patients receiving care at ACO participant organizations and potential overlap in care redesign strategies adopted under the 2 payment models.

**Objective:**

To examine whether simultaneous participation in a Medicare Shared Savings Program (MSSP) ACO affects the association between hospitals’ participation in LEJR episodes under the Bundled Payments for Care Improvement (BPCI) initiative and patient outcomes compared with participation in the BPCI initiative alone.

**Design, Setting, and Participants:**

This cohort study, conducted from January 1 to May 31, 2019, used 2011 to 2016 Medicare claims data and incorporated an instrumental variable with a difference-in-differences method among 483 008 fee-for-service Medicare beneficiaries undergoing LEJR surgery at 212 bundled payment participant hospitals, 105 coparticipant hospitals, and 1413 nonparticipant hospitals in the United States.

**Exposures:**

Hospital participation in both the BPCI initiative and the MSSP (coparticipants), BPCI only (bundled payment participants), or neither (nonparticipants).

**Main Outcomes and Measures:**

Changes in clinical outcomes and mean LEJR episode spending.

**Results:**

A total of 483 008 patients (mean [SD] age, 73.0 [8.4] years; 308 173 [63.8%] female) were included in the study. No differential changes were found in patient and hospital characteristics across participation groups. In adjusted analysis, coparticipants had 1.5% (95% CI, 0.7%-2.2%; *P* < .001) more unplanned readmissions than did bundled payment participants. Compared with bundled payment participants, coparticipants also had differentially greater decreases in hospital length of stay (adjusted difference-in-differences value, −5.3%; 95% CI, −7.1% to −3.5%; *P* < .001) and home health care use (adjusted difference-in-differences value, −3.4%; 95% CI, −4.5% to −2.3%; *P* < .001) and greater increases in postdischarge outpatient follow-up (adjusted difference-in-differences value, 2.1%; 95% CI, 0.9%-3.3%; *P* < .001). Coparticipants and bundled payment participants did not have differential changes in episode spending (adjusted difference-in-differences value, 0.4%; 95% CI, −0.7% to 1.6%; *P* = .46), although both groups had more decreased spending compared with nonparticipants.

**Conclusions and Relevance:**

Among bundled payment participants, coparticipation in ACOs was not associated with LEJR episode savings but was associated with differential changes in postacute care use patterns and unplanned readmissions. These findings support the longer-term benefits of LEJR bundles and suggest that coparticipants may adopt care redesign strategies that differ from hospitals with bundled payments only.

## Key Points

**Question:**

For hospitals participating in bundled payments for joint replacement surgery, what is the association between simultaneous participation in accountable care organizations and joint replacement outcomes?

**Findings:**

In a cohort study of 483 008 Medicare fee-for-service beneficiaries, compared with participation in joint replacement bundled payments alone, coparticipation was not associated with differential changes in episode spending. However, coparticipation in accountable care organizations was associated with differentially greater decreases in hospital length of stay and home health care use, greater increases in postdischarge outpatient follow-up, and smaller reductions in unplanned readmissions.

**Meaning:**

Hospitals coparticipating in accountable care organizations and joint replacement bundled payments may adopt different care redesign strategies from hospitals in bundled payments alone without differences in episode spending.

## Teaser

This cohort study examines the association between coparticipation in voluntary accountable care organizations (ACOs) and hospital performance on lower-extremity joint replacement outcomes and spending.

## Introduction

On the basis of early savings from the Bundled Payments for Care Improvement (BPCI) initiative,^1,2,3^ Medicare has continued to scale voluntary lower-extremity joint replacement (LEJR) bundles among hospitals across the United States. In particular, early experience in BPCI model 2 (through which hospitals achieved a mean of 3%-4% LEJR episode savings)^2,3^ was used directly to design a successor program, BPCI-Advanced. Since launching in October 2018, BPCI-Advanced has engaged 715 hospitals around the country and become the largest bundled payment program to date.^4^

Concurrently, an increasing number of hospitals have participated in voluntary accountable care organizations (ACOs) through initiatives such as the Medicare Shared Savings Program (MSSP).^5,6,7^ Despite different emphases, with bundled payments focusing on outcomes for episodes of care starting with hospitalization and ACOs focusing on outcomes during a year across all settings, the payment models share the goal of containing costs while maintaining or improving quality of care. For example, participation in both payment models is associated with lower spending on postacute care (personal communication: Navathe AS, Emanuel EJ, Venkataramani AS, Huang Q, Gupta A, Dinh CT, Shan EZ, Small D, Coe NB, Wang E, Ma X, ZHu J, Cousins DS, Liao JM; 2019).^2,8,9^ As a common and major driver of health care use and spending, LEJR is a highly relevant target for both bundled payments (the most commonly selected episode across existing programs) and ACOs (prevalent procedure performed on 3% of all Americans >60 years of age).^10^

Medicare program rules permit hospitals bundling LEJR to simultaneously participate (ie, coparticipate) in ACOs. Although early ACOs have not necessarily emphasized or contained surgical spending, 20% of surgeons participate in at least 1 ACO, and hospital initiatives implemented in response to ACO participation can nonetheless affect the care of surgical patients.^11,12,13^ Moreover, ACO incentives to manage total costs of care inherently include surgical spending, which can account for up to 50% of hospital expenditures and 30% of total health care spending.^12^ Therefore, coparticipation could promote better payment model performance if organizational changes under ACOs synergize with practice redesign under LEJR bundles. For instance, because both payment models include an emphasis on postacute care use, initiatives adopted by hospitals under both models (eg, shifting discharge away from institutional postacute care facilities, strengthening transitions of care programs) could promote greater performance if invested in and implemented concurrently.

Conversely, coparticipation could lead to no additional benefit or even weaken performance by limiting hospitals’ ability to redesign care to promote performance under LEJR bundles. In particular, hospitals that try to simultaneously implement different programs for LEJR bundles and ACOs may risk underinvesting in both by spreading resources or staff time too thinly across them. Hospitals that seek to align programs to serve the needs of both payment models may create redundancy or confusion when engaging different practitioners and operational teams in related yet distinct activities (eg, redesigning discharge processes for patients undergoing LEJR surgery while addressing care transitions for high-risk ACO patients with multiple chronic conditions).

However, despite broad participation in both payment models and the recognition of the potential for positive or negative interactions,^14,15^ it remains unknown how outcomes are affected for patients receiving care from voluntary bundled payment participants that also coparticipate in ACOs. Understanding the effect of coparticipation is of policy and clinical interest as policy makers continue expanding both payment models and health care organizations face the prospect of participating in one at a time or both simultaneously. There is a particular need for insight about longer-term outcomes given the duration of BPCI-Advanced (a 5-year program running through 2023) and that benefits from payment models may require several years to be realized.^5,6^ Furthermore, evaluations conducted by Medicare contractors focus on the effects of single payment models but not interactions between multiple payment models. Therefore, using LEJR as a relevant example, we evaluated the association of coparticipation in voluntary ACOs with hospital performance on LEJR bundle outcomes and spending.

## Methods

### Study Overview

From January 1 to May 31, 2019, we used 2011 to 2016 Medicare claims data to conduct a cohort study comparing 3-year changes in clinical outcomes and spending at hospitals participating in both LEJR bundles under the BPCI initiative and an MSSP ACO compared with changes at hospitals participating in only LEJR bundles and hospitals participating in neither program. We used an instrumental variable together with a difference-in-differences method^16,17^ to account for evidence that performance in voluntary payment models may be associated with shifts in unobservable patient case mix (eg, shifts based on characteristics such as poor social support, which are recognizable to hospital participants but unobservable in data) rather than with actual care improvements.^18^ The University of Pennsylvania Institutional Review Board approved this study with a waiver of informed consent because the retrospective nature made seeking informed consent infeasible and there was minimal risk to study participants. Data were not fully deidentified. Our study followed guidelines from the Strengthening the Reporting of Observational Studies in Epidemiology (STROBE) reporting guideline.^19^

### Study Periods, Hospitals, and Markets

We defined baseline (prebundled payment; January 1, 2012, to September 30, 2013) and intervention (bundled payment; October 1, 2013, to September 30, 2016) periods. We identified hospitals participating in LEJR bundles under BPCI model 2 and the MSSP ACO program using participant lists from Medicare.^1^ In particular, we identified hospitals participating in LEJR bundles under BPCI model 2 by compiling publicly available BPCI participant lists and defining BPCI hospitals on a quarterly basis. This approach reflected both the time-varying nature of BPCI program enrollment and accommodated the potential for hospital entry into and exit from the program over time.

We excluded non–acute care hospitals, those that did not participate in Medicare’s inpatient prospective payment system, and those with low LEJR volume among other exclusions (eFigure in the Supplement). Eligible hospitals were categorized as participating in BPCI only (bundled payment participants), both BPCI and MSSP (coparticipants), or neither (nonparticipants). Analogously, we used hospital referral regions^20^ to define coparticipant, bundled payment participant, or nonparticipant markets based on whether they contained at least 1 corresponding hospital type. Hospital referral regions with both coparticipants and bundled payment participants were defined as coparticipant markets.

We did not examine hospitals in ACOs only because payment model incentives apply only to ACO-attributed patients receiving LEJR surgery, potentially leading to unfair comparisons with hospitals in bundled payments for whom incentives apply to all patients undergoing LEJR surgery. Given our focus on voluntary programs, we also excluded hospitals participating in Medicare’s mandatory Comprehensive Care for Joint Replacement Model from our analysis. Hospital characteristics were obtained through data from Medicare claims, the American Hospital Association annual survey, Hospital Compare, and the Centers for Medicare & Medicaid Services Improving Medicare Post–Acute Care Transformation (IMPACT) file.^21,22,23^

### Patients and Episode Construction

We identified patients hospitalized across all 3 hospital groups between 2011 and 2016 for major hip and knee joint replacement or reattachment of lower extremity with and without major complications or comorbid conditions (Medicare Severity–Diagnosis Related Groups 469 and 470). We used a 100% sample of patients hospitalized through the BPCI initiative and a 20% national sample of patients hospitalized through non-BPCI hospitals, excluding individuals who lacked continuous Medicare fee-for-service enrollment during a qualifying LEJR episode or during a 180-day lookback period. We also excluded patients with end-stage renal disease, those with overlapping or repeating episodes, and those who died during hospitalization (eFigure in the Supplement). To increase sample homogeneity,^2,8^ we also excluded individuals younger than 65 years and older than 90 years and patients receiving hospice care. Medicare claims were used to construct episodes spanning from hospitalization for Medicare Severity–Diagnosis Related Group 469 or 470 through 90 days after hospital discharge using methods described previously.^8,21,24^ We used a number of patient characteristics to control for baseline health, including demographics, Medicare and Medicaid dual eligibility status, Elixhauser comorbidities,^25^ and prior health care use.

### Outcomes

Primary clinical outcome measures included 90-day risk-standardized mortality, unplanned readmission, and emergency department visit rates as well as LEJR-specific complication rates.^26^ Secondary clinical outcomes included index hospitalization length of stay, 30-day and 60-day unplanned readmissions, discharge to a home health agency (HHA), discharge to an institutional postacute care facility (skilled nursing facility or inpatient rehabilitation facility), and postdischarge follow-up (outpatient office visit within 7 days of discharge from hospital or an institutional postacute care facility). The spending outcome was the mean spending (ie, Medicare payment) per LEJR episode. In line with prior methods, spending estimates were standardized and transformed into 2016 US dollars.^21,22,23,27,28^

### Statistical Analysis

We compared patient and hospital characteristics across groups. We used the χ^2^ and unpaired *t* tests to compare categorical variables and a combination of unpaired *t* tests, Kruskal-Wallis, and Wilcoxon rank sum tests to compare continuous variables.

We used a difference-in-differences approach to estimate differential changes in outcomes among patients admitted across hospital groups in the prebundled payment and bundled payment periods.^29,30^ Clinical outcomes were analyzed with ordinary least squares regression, whereas episode spending was analyzed using generalized linear models with a log link and γ distribution. Models included hospital and quarter fixed effects; controlled for market characteristics, patient demographics, and clinical conditions (eTable 1 in the Supplement); and used bootstrapped SEs.^31,32^ We also tested the parallel trends assumption for our difference-in-differences method.

Prior evidence^18^ suggests that the BPCI initiative may be susceptible to patient selection because of unobservable characteristics (ie, changes in the types of patients receiving care under bundled payments after organizations begin participation in BPCI based on patient characteristics not captured in claims data). For example, physicians in BPCI with operating privileges at multiple hospitals preferentially selected lower-risk patients to undergo surgery at BPCI hospitals and higher-risk patients to undergo surgery at non-BPCI hospitals^18^ based on characteristics unobservable in data. Because some physicians who perform LEJR nationwide retain operating privileges at multiple hospitals, such selective referrals highlight the potential for patient selection and the need for analyses that account for this factor. We addressed the potential for this and other forms of patient selection using an instrumental variable approach in a 2-stage least squares regression (eMethods 1 and eTable 2 in the Supplement).

Specifically, the instrumental variable used historical hospital referral patterns in 2011, before BPCI began, to calculate the probability of LEJR hospitalization at an eventual bundled payment participant. In the first-stage regression, we used probabilities as an instrument for actual LEJR hospitalization at BPCI participants. In the second stage, we assessed the association between instrumented admission to a bundled payment participant and study outcomes. Although selection based on unobservable characteristics was possible before hospital participation in the BPCI initiative, referral patterns in that baseline period could not explain changes in patient selection occurring as a result of subsequent program participation. Therefore, our approach mitigated the effects of patient selection based on unobservable patient characteristics that occurred after hospitals started participating in the BPCI initiative.

Statistical tests were 2-tailed, with robust SEs corrected for heteroscedasticity.^33^ A Bonferroni-adjusted α = .01 was used for significance of primary clinical and spending outcomes. Secondary clinical outcomes were considered to be significant at α = .05 without adjustment for multiple testing. Analyses were performed using SAS statistical software, version 9.4 (SAS Institute Inc) or Stata software, version 15.1 (StataCorp LLC).

To evaluate the extent to which changes in primary clinical and spending outcomes were associated with patient selection, we repeated our difference-in-differences analyses without an instrumental variable. In addition, we tested to the robustness of our results using analyses that excluded the time between January and September 2013 (to account for any anticipatory changes that hospitals may have initiated in the quarters leading up to the beginning of BPCI) and used an intention-to-treat approach in assigning hospital BPCI participation (which defined BPCI status based on participation at any point without consideration of subsequent program exit).

## Results

Our sample included 483 008 fee-for-service Medicare beneficiaries (mean [SD] age, 73.0 [8.4] years; 308 173 [63.8%] female) who underwent LEJR surgery at 212 bundled payment hospitals, 105 coparticipant hospitals, and 1413 nonparticipant hospitals nationwide. Wald tests did not indicate divergent secular trends overall among hospital groups during the prebundled payment period (eMethods 2 in the Supplement).

### Patient, Hospital, and Market Characteristics

A number of patient characteristics differed across hospital groups (Table 1). For example, hospital groups differed with respect to the proportion of black, female, and dual-eligible patients as well as patients with prior acute care hospital and inpatient rehabilitation facility use. Patient characteristics varied by study period for each hospital group, but no meaningful differential trends were found for patients across groups (ie, those receiving care at coparticipants vs bundled payment participants and nonparticipants) (eTable 3, eTable 4, and eTable 5 in the Supplement).

**Table 1.** Patient Characteristics by Participation Status, 2012-2016^a^

Characteristic	Coparticipants (n = 103 737)	Bundled Payment Participants (n = 176 513)	Nonparticipants (n = 202 758)	*P* Value
Sample characteristics^**b**^				
Markets	60 (14.1)	103 (24.1)	264 (61.8)	NA
Hospitals	105 (6.1)	212 (12.3)	1413 (81.7)	NA
Episodes	103 737 (21.5)	176 513 (36.5)	202 758 (42.0)	NA
Patient characteristics				
Age, mean (SD), y	73.0 (8.3)	73.1 (8.4)	73.0 (8.5)	<.001
Black race^c^	5760 (5.6)	13 034 (7.4)	11 256 (5.6)	<.001
Female	66 571 (64.2)	113 621 (64.4)	127 981 (63.1)	<.001
Dual eligible^d^	9990 (9.6)	20 544 (11.6)	25 327 (12.5)	<.001
Elixhauser comorbidity index score, mean (SD)^e^^,^^f^	4.2 (10.4)	4.5 (10.5)	4.6 (10.7)	<.001
Prior use^f^				
Acute care hospital	15 735 (15.2)	26 944 (15.3)	34 858 (17.2)	<.001
IRF	1338 (1.3)	2222 (1.3)	2797 (1.4)	.004
SNF	4290 (4.1)	7188 (4.1)	9180 (4.5)	<.001
Market characteristics, median (IQR)				
Quarterly LEJR volume	456 (238-751)	321 (175-594)	227 (129-440)	<.001
Hospital beds	3708 (2028-8593)	3259 (1458-6266)	2162 (1217-3919)	<.001
SNF beds	6998 (3973-13 119)	5244 (2775-9389)	3586 (2033-7258)	<.001
Penetration, mean (SD), %				
MA	26.1 (11.4)	29.5 (12.8)	26.4 (13.2)	.09
ACO	17.1 (7.9)	11.5 (6.9)	10.2 (7.7)	<.001
HHI score, mean (SD)				
Hospital	2213.2 (1713.9)	2652.4 (1806.7)	3183.0 (2023.1)	<.001
SNF	799.8 (513.2)	1181.2 (894.7)	1470.7 (1094.8)	<.001
PGP market	37 (61.7)	57 (55.3)	124 (47.0)	.07

Abbreviations: ACO, accountable care organization; HHI, Herfindahl-Hirschman Index; IQR, interquartile range; IRF, inpatient rehabilitation facility; LEJR, lower-extremity joint replacement; MA, Medicare Advantage; NA, not applicable; PGP, physician group practice; SNF, skilled nursing facility.

^a^ Data are presented as number (percentage) of patients unless otherwise indicated.

^b^ Characteristics for coparticipant and bundled payment participant patients were drawn from a 100% Medicare claims sample, whereas characteristics for nonparticipant patients were drawn from a 20% Medicare claims sample.

^c^ Race was divided as black vs others because of existing disparities in access to LEJR among black patients specifically.

^d^ Dual eligible indicates eligibility for both the Medicare and Medicaid programs as an indicator of low socioeconomic status.

^e^ The Elixhauser comorbidity score is an index of severity with a range of −32 to 92, with increasing scores correlated with increased probability of in-hospital death.

^f^ Calculated using data from the year before LEJR hospitalization.

Hospital groups also varied in several hospital characteristics (Table 2). In particular, coparticipant hospitals were larger (median number of beds, 303 vs 251 beds for bundled payment participants and 145 beds for nonparticipants; *P* < .001) with greater market share (9.2% vs 6.7% for bundled payment participants and 4.8% for nonparticipants; *P* < .001). Compared with other hospital groups, coparticipants were also more likely to be urban, not-for-profit teaching hospitals.

**Table 2.** Hospital Characteristics by Participation Status, 2011^a^

Characteristic	Coparticipants (n = 105)	Bundled Payment Participants (n = 212)	Nonparticipants (n = 1413)	*P* Value^b^
Characteristics of hospital admissions				
Annual admissions for top-10 BPCI episodes, mean (SD), %^c^	23.0 (5.6)	21.6 (4.6)	25.5 (7.1)	<.001
Annual admissions for LEJR, median (IQR)^d^	206 (117-388)	146 (81-268)	90 (41-182)	<.001
Proportion of discharges to highest-volume IRF, median (IQR), %^d^	78.9 (0.0-98.7)	79.6 (10.2-100)	50.0 (0.0-100)	.002
Proportion of discharges to highest-volume SNF, mean (SD), %	27.2 (16.0)	28.4 (17.6)	38.6 (20.4)	<.001
90-Day readmission rate, median (IQR)^d^	11.1 (9.0-14.7)	11.7 (9.1-15.6)	11.1 (5.9-18.2)	.08
90-Day LEJR episode spending, median (IQR), $^d^	23 903 (21 194-26 124)	25 096 (22 723-28 512)	23 517 (20 350-28 097)	<.001
Characteristics of hospital organization				
No. of beds, median (IQR)^d^	303 (172-415)	251 (163-391)	145 (81-253)	<.001
Ownership status				
For profit	3 (2.9)	54 (25.8)	338 (24.6)	<.001
Not for profit	100 (96.2)	144 (68.9)	818 (59.4)	
Government	1 (1.0)	11 (5.3)	221 (16.1)	
Member of a system	80 (76.9)	171 (81.8)	839 (60.9)	<.001
Teaching status^e^				
Major	18 (17.3)	28 (13.4)	98 (7.1)	<.001
Minor	45 (43.2)	68 (32.5)	335 (24.3)	
Nonteaching	41 (39.4)	113 (54.1)	944 (68.6)	
Intern and resident to bed ratio, median (IQR)^d^	0.0 (0.0-0.1)	0.0 (0.0-0.06)	0.0 (0.0-0.02)	<.001
Disproportionate share hospital payments, median (IQR), $^f^	2 656 317 (364 284-6 101 116)	2 742 670 (492 948-7 598 080)	1 238 117 (427 915-4 005 455)	<.001
Urban status	104 (100)	207 (99.0)	1278 (92.8)	<.001
Medicare days (as percentage of total patient days), mean (SD)	50.3 (10.7)	52.4 (10.7)	51.0 (13.9)	.29
Market share, median (IQR), %^d^	9.2 (4.5-17.6)	6.7 (2.7-18.4)	4.8 (1.8-12.0)	<.001

Abbreviations: BPCI, Bundled Payments for Care Improvement; IQR, interquartile range; IRF, inpatient rehabilitation facility; LEJR, lower-extremity joint replacement; SNF, skilled nursing facility.

^a^ Data are presented as number (percentage) of hospitals unless otherwise indicated.

^b^ Kruskal-Wallis tests were used to test the differences in continuous variables and χ^2^ tests for categorical variables.

^c^ Major joint replacement of the lower extremity; double joint replacement of the lower extremity; revision of the hip or knee; hip and femur procedures except major joint; lower extremity and humerus procedure except hip, foot, and femur; coronary artery bypass graft; acute myocardial infarction; congestive heart failure; simple pneumonia and respiratory infections; chronic obstructive pulmonary disease; and bronchitis or asthma.

^d^ Median (IQR) provided where data are skewed.

^e^ From the American Hospital Association Annual Survey, major teaching hospitals are those that are members of the Council of Teaching Hospitals (COTH), minor teaching hospitals are non-COTH members that had a medical school affiliation reported to the American Medical Association, and nonteaching hospitals are all other institutions.

^f^ Disproportionate share hospital payment percentage derived from the fiscal year 2017 Centers for Medicare & Medicaid Services Improving Medicare Post–Acute Care Transformation (IMPACT) file.

Market groups differed with respect to numerous characteristics (Table 1). For example, coparticipant markets had the highest LEJR volume (median, 456 quarterly procedures vs 321 among bundled payment participant markets and 227 among nonparticipant markets; *P* < .001) and hospital and skilled nursing facility bed supply. With the exception of LEJR volume, market characteristics did not differ across market groups (eTable 3, eTable 4, and eTable 5 in the Supplement).

### Clinical Outcomes

**Figure 1.**
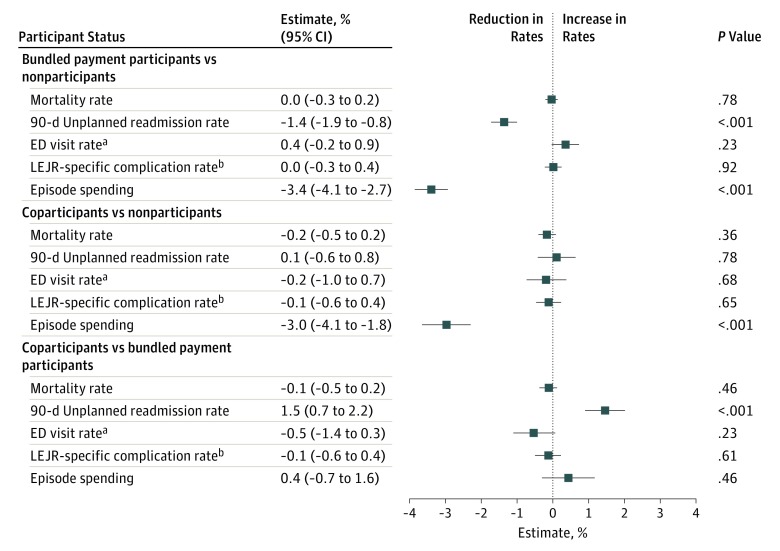
Adjusted Changes in Primary Clinical Outcomes and Episode Spending Associated With Participation Status, 2012-2016 Results from difference-in-differences models evaluating the association between participation status (coparticipation vs bundled payment participation, coparticipation vs nonparticipation) and differential changes in primary clinical outcomes and spending. Negative estimates indicate reductions in rates (ie, quality improvements). LEJR indicates left-extremity joint replacement. ^a^Emergency department (ED) visits without hospitalization. ^b^Defined by Hospital Compare.^26^

In unadjusted analysis, secular trends were observed across hospital groups for multiple outcomes (eTable 6, eTable 7, and eTable 8 in the Supplement). In adjusted analyses using the instrumental variable (Figure 1), bundled payment participants and coparticipants did not differ during 3 years with respect to 90-day risk-standardized mortality (adjusted difference-in-differences [aDiD] value, −0.1%; 95% CI, −0.5% to 0.2%; *P* = .46), unplanned emergency department visits (aDiD value, −0.5%; 95% CI, −1.4% to 0.3%; *P* = .23), or LEJR complications (aDiD value, −0.1%; 95% CI, −0.6% to 0.4%; *P* = .61). However, coparticipants had differentially smaller reductions in readmissions (aDID value, 1.5%; 95% CI, 0.7%-2.2%; *P* < .001) than did bundled payment participants.

Compared with nonparticipants, both bundled payment participants and coparticipants had comparable mortality, unplanned emergency department visits, and LEJR complication rates (Figure 1). Although coparticipants and nonparticipants also had comparable unplanned 90-day readmissions, bundled payment participants had differentially greater reductions in readmissions (aDID value, −1.4%; 95% CI, −1.9% to −0.8%; *P* < .001) than did nonparticipants.

**Figure 2.**
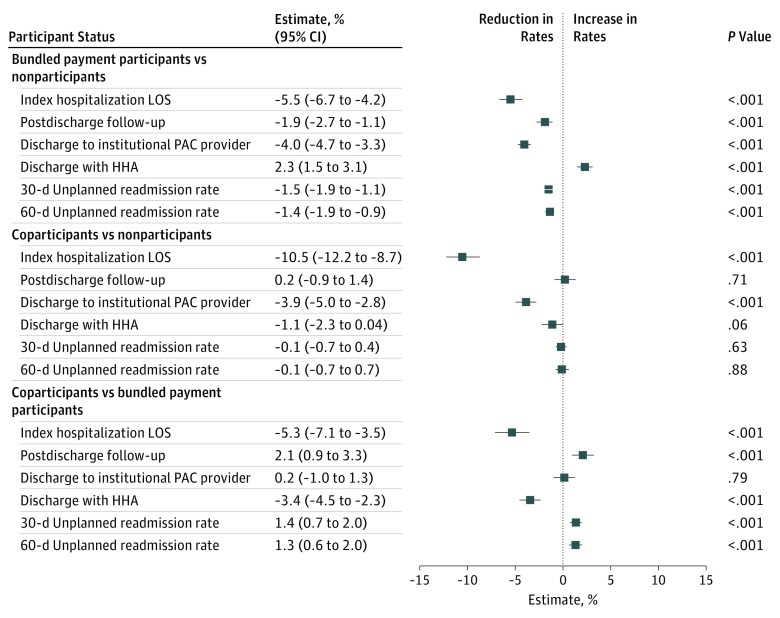
Adjusted Changes in Secondary Clinical Outcomes Associated With Participation Status, 2012-2016 Results from difference-in-differences models evaluating the association between participation status (coparticipation vs bundled payment participation, coparticipation vs nonparticipation) and differential changes in secondary clinical outcomes. Negative estimates indicate reductions in rates (ie, quality improvements). Institutional postacute care (PAC) providers were skilled nursing facilities or inpatient rehabilitation facilities. HHA indicates home health agency; LOS, length of stay.

Hospital groups also differed with respect to a number of secondary clinical outcomes in unadjusted comparisons (eTable 6, eTable 7, and eTable 8 in the Supplement). In adjusted analyses (Figure 2), compared with bundled payment participants, coparticipants had differentially greater reductions in index hospitalization length of stay (aDiD value, −5.3%; 95% CI, −7.1% to −3.5%) and HHA use (aDID value, −3.4% [95% CI, −4.5% to −2.3%] for hospital discharge with HHA services) and differentially greater increases in postdischarge follow-up (aDiD value, 2.1%; 95% CI, 0.9%-3.3%), 30-day unplanned admissions (aDiD value, 1.4%; 95% CI, 0.7%-2.0%), and 60-day unplanned admissions (aDiD value, 1.3%; 95% CI, 0.6%-2.0%) (*P* < .001 for all variables).

Compared with nonparticipants, both coparticipants and bundled payment participants had differentially greater reductions in length of stay and institutional postacute care facility use. Bundled payment participants had differential increases in postdischarge follow-up and decreases in 30- and 60-day readmissions compared with nonparticipants.

### Episode Spending

For patients at coparticipant hospitals, mean (SD) episode spending was $23 142 ($14 257) in the prebundled payment period and $21 657 ($12 720) in the bundled payment period (difference, −$1485; *P* < .001) (eTable 6 and eTable 7 in the Supplement). Mean (SD) episode spending was $24 298 (14 167) in the prebundled payment period and $22 650 ($14 009) in the bundled payment period among patients at bundled payment participant hospitals (difference, −$1648; *P* < .001) and $23 145 ($14 140) in the prebundled payment period and $22 413 ($13 883) in the bundled payment period among patients at nonparticipant hospitals (difference, −$732; *P* < .001). Unadjusted differential changes in raw mean episode spending was −9.9% (−$163; *P* = .14) between coparticipants and bundled payment participants and 103% (−$753; *P* < .001) between coparticipants and nonparticipants (eTable 6 and eTable 7 in the Supplement).

In adjusted instrumental variable analyses (Figure 1), no statistically significant differential changes were found between coparticipants and bundled payment participants in mean episode spending (aDiD value, 0.4%, 95% CI, −0.7% to 1.6%; *P* = .46). Compared with nonparticipants, episode spending decreased more among coparticipants (aDiD value, −3.0%; 95% CI, −4.1% to −1.8%) and bundled payment participants (aDiD value, −3.4%; 95% CI, −4.1% to −2.7%) (*P* < .001 for both).

### Sensitivity Analyses

In adjusted analyses without the instrumental variable (eTable 9 in the Supplement), findings were qualitatively similar to those from main analyses overall. The direction of differential changes in unplanned 90-day readmissions was similar between sensitivity and primary analyses for comparisons of coparticipants vs bundled payment participants (1.1% higher vs 1.5% higher in analyses using the instrumental variable) as well as comparisons of bundled payment participants and nonparticipants (0.7% lower vs 1.4% lower in analyses using the instrumental variable). However, comparisons for readmissions did not achieve statistical significance in sensitivity analyses. Results from other sensitivity analyses were also qualitatively similar to those from our main analyses (eTable 10 and eTable 11 in the Supplement).

## Discussion

To our knowledge, this was the first study to evaluate changes in clinical outcomes and spending for LEJR bundles in the context of simultaneous ACO and bundled payment participation. Our study found that coparticipants and bundled payment participants did not have differential changes in episode spending and that both groups had differentially greater decreases in spending than nonparticipants without decrements in clinical outcomes. However, compared with bundled payment participation alone, simultaneous ACO and bundled payment participation was associated with a smaller differential reduction in readmissions and differential changes in postdischarge care. These findings have 3 important policy implications in light of ongoing nationwide expansion of both payment models.

First, the favorable association of voluntary LEJR bundles with episode spending is underscored by the lower spending observed during 3 years among both bundled payment participants and coparticipants compared with nonparticipants. Policy makers may be reassured by these findings, which suggest that LEJR episode savings are durable over time and that coparticipation arising in part from expansion of ACO participation nationwide may not be associated with reduced LEJR episode savings.

Second, differential changes in unplanned readmission rates across participation groups may suggest that bundled payment participants and coparticipants adopt different clinical redesign strategies. In particular, our results demonstrated a well-known strategy for performance in LEJR bundles: shift discharges away from high-intensity postacute care facilities, such as skilled nursing facilities and inpatient rehabilitation facilities, and toward HHA.^2,8^ The comparative changes in hospital length of stay, HHA use, and postdischarge outpatient follow-up among coparticipants could suggest use of ACO infrastructure elements that focus on ambulatory care, reductions in inpatient and HHA use, and increases in close outpatient follow-up use.^34^

The association between coparticipation and differential changes in readmissions is noteworthy because both coparticipants and bundled payment–only participants had improvements in their readmission rates, but rates at coparticipants improved more slowly. Current Medicare policy is not designed to encourage coparticipation in bundled payments and other payment models, such as ACOs. In particular, when individual organizations participate in both bundled payments and ACOs, the agency assigns costs and implements financial incentives using accounting approaches that ultimately discourage coparticipation.^15^ However, some prior work^35^ has suggested that participation in multiple value-based programs is associated with better performance in Medicare's Hospital Readmissions Reduction Program. Therefore, although readmissions represent 1 of several primary outcomes and additional work is needed to identify factors associated with differential changes, our study used rigorous methods, a specific payment program, and a high-volume clinical episode to provide new insight and raise the possibility that coparticipation could be associated with unintended slowing of improvement in readmission rates.

Third, differences in results generated from analyses with or without the instrumental variable support the concern that voluntary bundled payments are susceptible to patient selection (ie, changes in outcomes associated with changes in the types of patients who undergo LEJR surgery) and emphasize the need to account for selection in future payment model evaluations. Our results also appear to provide some reassurance that such selection does not fully account for clinical or spending benefits in LEJR bundles.

### Limitations

This study has limitations. First, as a cohort study rather than a randomized trial of voluntary bundled payments, findings could be subject to residual confounding and patient and/or hospital selection. However, we mitigated these concerns through the quasi-experimental design, an instrumental variable, and use of hospital fixed effects and a set of patient and hospital characteristics. Collectively, these measures helped overcome limitations of prior voluntary LEJR bundle evaluations, mitigating the confounding, hospital selection (based on unobservable time-invariant factors), and patient selection with study results. Second, findings may not generalize from LEJR to medical condition episodes. Third, although we focused our study on BPCI model 2, it was the largest BPCI model by enrollment and the direct basis for BPCI-Advanced. Fourth, results from our instrumental variable analyses apply only to beneficiaries who underwent LEJR surgery at BPCI hospitals regardless of the policy, not to all beneficiaries receiving the procedure. Fifth, this study did not examine the effect of coparticipation among physician group practice participants. Sixth, although our analyses did not include hospitals participating in only ACOs, our focus was providing valid comparisons of changes under incentives focused specifically on LEJR rather than all conditions and procedures. Seventh, our findings may not generalize to non-Medicare patients undergoing LEJR surgery. Eighth, the study findings may not apply to mandatory programs, such as the Comprehensive Care for Joint Replacement model, particularly because major program features and characteristics of participating hospitals differ.^21^

## Conclusions

Among bundled payment participants, concurrent participation in ACOs was not associated with additional LEJR episode savings but was associated with changes in postacute care use patterns and more unplanned readmissions. Our findings suggest the potential benefits of voluntary LEJR bundles, that coparticipants and bundled payment participants may adopt different clinical redesign strategies, and that patient selection may be associated with bundled payment evaluations.

## Notes

Supplement. **eFigure.** Study Sample Flow Diagram **eTable 1.** Variables Used in Statistical Models **eTable 2.** Balance in Patient Characteristics Between Hospital Groups by Values of the Instrumental Variable **eTable 3.** Patient and Market Characteristics by Participation Status (Coparticipant Patients vs Bundled Payment Participant Patients) and Study Period, 2012-2016 **eTable 4.** Patient and Market Characteristics by Participation Status (Coparticipant Patients vs Nonparticipant Patients) and Study Period, 2012-2016 **eTable 5.** Patient and Market Characteristics by Participation Status (Bundled Payment Participant Patients vs Nonparticipants) and Study Period, 2012-2016 **eTable 6.** Unadjusted Changes in Clinical Outcomes and Spending Associated With Participation Status (Coparticipants vs Bundled Payment Participants), 2012-2016 **eTable 7.** Unadjusted Changes in Clinical Outcomes and Spending Associated With Participation Status (Coparticipants vs Nonparticipants), 2012-2016 **eTable 8.** Unadjusted Changes in Clinical Outcomes and Spending Associated With Participation Status (Coparticipants vs Nonparticipants), 2012-2016 **eTable 9.** Sensitivity Analyses Without Use of an Instrumental Variable **eTable 10.** Sensitivity Analyses Excluding January-September 2013 **eTable 11.** Sensitivity Analyses Using an Intention-to-Treatment Approach to Assigning Hospital BPCI Status **eMethods 1.** Instrumental Variable Approach **eMethods 2.** Tests of Parallel Trends Between Hospital Groups for Primary Clinical Outcomes and Spending Variables
