# Supplementary material for: Association of Bundled Payments for Joint Replacement Surgery and Patient Outcomes With Simultaneous Hospital Participation in Accountable Care Organizations
Source: JAMA Netw Open. 2019 Sep 27;2(9):e1912270. doi: 10.1001/jamanetworkopen.2019.12270 (PMC6777392; doi:10.1001/jamanetworkopen.2019.12270)
Supplement: Supplement. — eFigure. Study Sample Flow Diagram eTable 1. Variables Used in Statistical Models eTable 2. Balance in Patient Characteristics Between Hospital Groups by Values of the Instrumental Variable eTable 3. Patient and Market Characteristics by Participation Status (Coparticipant Patients vs Bundled Payment Participant Patients) and Study Period, 2012-2016 eTable 4. Patient and Market Characteristics by Participation Status (Coparticipant Patients vs Nonparticipant Patients) and Study Period, 2012-2016 eTable 5. Patient and Market Characteristics by Participation Status (Bundled Payment Participant Patients vs Nonparticipants) and Study Period, 2012-2016 eTable 6. Unadjusted Changes in Clinical Outcomes and Spending Associated With Participation Status (Coparticipants vs Bundled Payment Participants), 2012-2016 eTable 7. Unadjusted Changes in Clinical Outcomes and Spending Associated With Participation Status (Coparticipants vs Nonparticipants), 2012-2016 eTable 8. Unadjusted Changes in Clinical Outcomes and Spending Associated With Participation Status (Coparticipants vs Nonparticipants), 2012-2016 eTable 9. Sensitivity Analyses Without Use of an Instrumental Variable eTable 10. Sensitivity Analyses Excluding January-September 2013 eTable 11. Sensitivity Analyses Using an Intention-to-Treatment Approach to Assigning Hospital BPCI Status eMethods 1. Instrumental Variable Approach eMethods 2. Tests of Parallel Trends Between Hospital Groups for Primary Clinical Outcomes and Spending Variables [file jamanetwopen-2-e1912270-s001.pdf]

Supplementary Online Content

Liao JM, Emanuel EJ, Venkataramani AS, et al. Association of bundled payments for joint replacement surgery and patient outcomes with simultaneous hospital participation in accountable care organizations. *JAMA Netw Open*. 2019;2(9): e1912270. doi:10.1001/jamanetworkopen.2019.12270

eFigure. Study Sample Flow Diagram

eTable 1. Variables Used in Statistical Models

eTable 2. Balance in Patient Characteristics Between Hospital Groups by Values of the Instrumental Variable

eTable 3. Patient and Market Characteristics by Participation Status (Coparticipant Patients vs Bundled Payment Participant Patients) and Study Period, 2012-2016

eTable 4. Patient and Market Characteristics by Participation Status (Coparticipant Patients vs Nonparticipant Patients) and Study Period, 2012-2016

eTable 5. Patient and Market Characteristics by Participation Status (Bundled Payment Participant Patients vs Nonparticipants) and Study Period, 2012-2016

eTable 6. Unadjusted Changes in Clinical Outcomes and Spending Associated With Participation Status (Coparticipants vs. Bundled Payment Participants), 2012-2016

eTable 7. Unadjusted Changes in Clinical Outcomes and Spending Associated With Participation Status (Coparticipants vs Nonparticipants), 2012-2016

eTable 8. Unadjusted Changes in Clinical Outcomes and Spending Associated With Participation Status (Bundled Payment Participants vs Nonparticipants), 2012-2016

eTable 9. Sensitivity Analyses Without Use of an Instrumental Variable

eTable 10. Sensitivity Analyses Excluding January-September 2013

eTable 11. Sensitivity Analyses Using an Intention-to-Treatment Approach to Assigning Hospital BPCI Status

eMethods 1. Instrumental Variable Approach

eMethods 2. Tests of Parallel Trends Between Hospital Groups for Primary Clinical Outcomes and Spending Variables

This supplementary material has been provided by the authors to give readers additional information about their work.

© 2019 Liao JM et al. *JAMA Network Open.*

eFigure. Study sample flow diagram

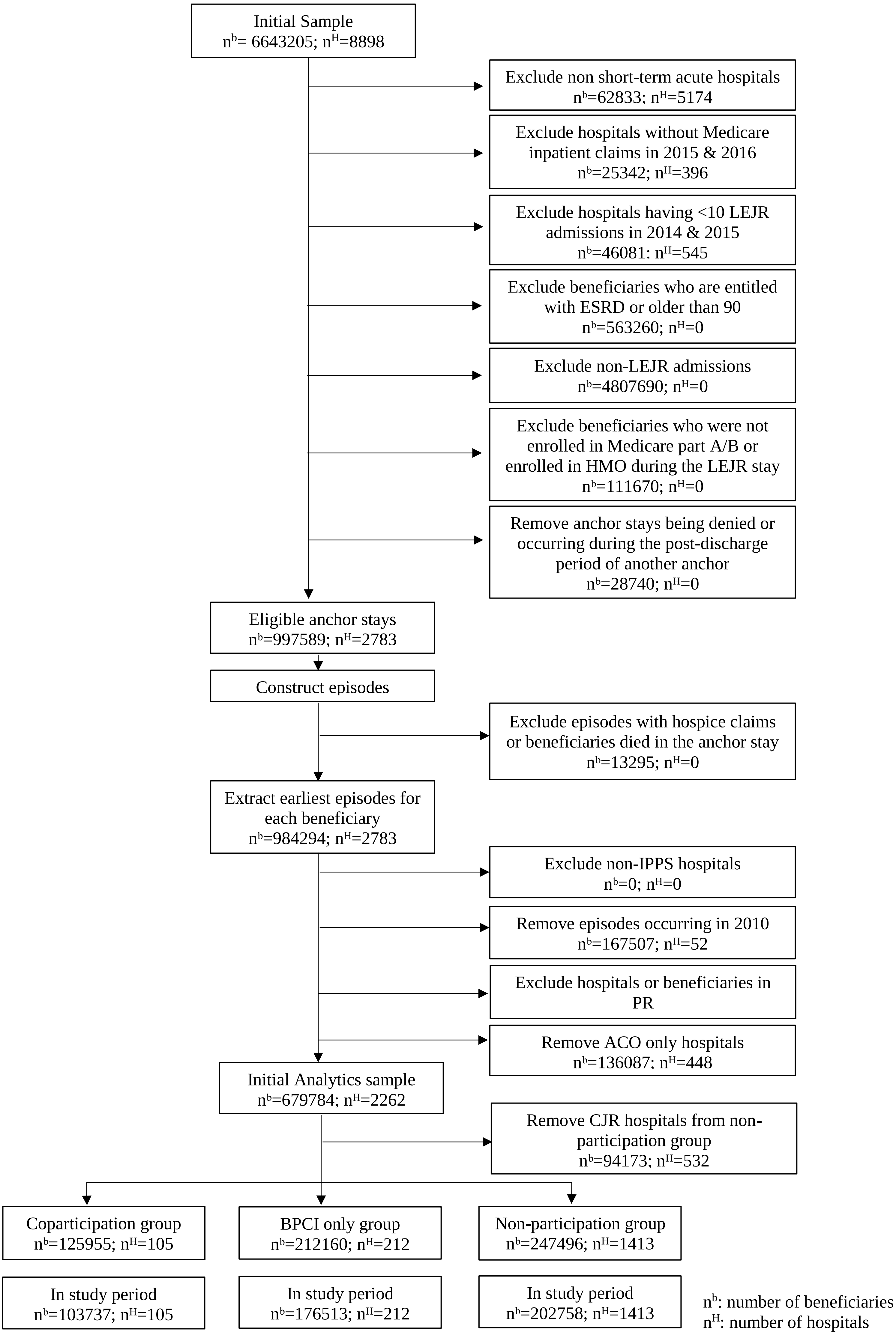

© 2019 Liao JM et al. *JAMA Network Open.*

eTable 1. Variables used in statistical models

**Patient Characteristics**
Sex
Race
Age
Medicare/Medicaid dual eligibility
Zip code
Index hospitalization DRG
Elixhauser Comorbidities
Congestive heart failure
Valvular disease
Pulmonary circulation disease
Peripheral vascular disease
Hypertension with complication
Paralysis
Other neurological disorders
Chronic pulmonary disease
Diabetes without chronic complications
Diabetes with chronic complications
Hypothyroidism
Renal failure
Liver disease
Peptic ulcer disease excluding bleeding
Acquired immune deficiency syndrome
Lymphoma
Metastatic cancer
Solid tumor without metastasis
Rheumatoid arthritis/collagen vascular disease
Coagulopathy
Obesity
Weight loss
Fluid and electrolyte disorders
Chronic blood loss anemia
Deficiency anemias
Alcohol abuse
Drug abuse
Psychoses
Depression
Acute care hospital use in prior 12 months
IRF use in prior 12 months
SNF use in prior 12 months
**Market characteristics**
ACO penetration based on physician attrition
MA penetration
HHI for hospital
HHI for SNF
Average hospital bed count
Presence of BPCI PGPs

© 2019 Liao JM et al. *JAMA Network Open.*

eTable 2. Balance in patient characteristics between hospital groups by values of the instrumental variable

Coparticipant vs bundled payment participant	Original Observed Data			IV Quartile 1			IV Quartile 2			IV Quartile 3			IV Quartile 4		
	CP	BP	SMD	CP	BP	SMD	CP	BP	SMD	CP	BP	SMD	CP	BP	SMD
**# of episodes**	65411	112345		2448	3915		11681	25728		25606	35313		25674	47375	
**Age**															
Mean	73.0	73.0	0.0	72.3	72.3	0.0	72.3	72.9	-0.1	73.0	72.9	0.0	73.2	73.3	0.0
SD	8.1	8.2		8.2	8.7		8.5	8.4		8.0	8.4		8.1	8.0	
**Elixhauser score**															
Mean	4.0	4.3	0.0	4.3	4.8	0.0	4.1	4.5	0.0	4.1	4.3	0.0	3.8	4.1	0.0
SD	10.3	10.5		10.9	11.0		10.3	10.8		10.4	10.6		10.1	10.2	
**Black, %**	5.5	7.3	-0.1	3.0	5.9	-0.1	6.4	5.7	0.0	5.1	8.4	-0.1	5.6	7.5	-0.1
**Female, %**	63.8	63.9	0.0	59.6	60.1	0.0	62.4	63.2	0.0	63.2	64.0	0.0	65.5	64.6	0.0
**Dual Eligible, %**	9.1	11.3	-0.1	9.1	11.3	-0.1	10.7	15.2	-0.1	8.5	12.0	-0.1	9.0	8.6	0.0
**Prior acute use, %**	14.6	14.6	0.0	14.4	15.1	0.0	15.8	15.6	0.0	14.7	15.3	0.0	13.9	13.4	0.0
**Prior IRF use, %**	1.3	1.2	0.0	1.5	1.3	0.0	1.3	1.3	0.0	1.3	1.4	0.0	1.2	1.0	0.0
**Prior SNF use, %**	4.0	3.9	0.0	4.3	4.9	0.0	4.0	4.2	0.0	4.1	4.1	0.0	3.9	3.6	0.0
**bundled payment participant vs non-participant**	**Original Observed Data**			**IV Quartile 1**			**IV Quartile 2**			**IV Quartile 3**			**IV Quartile 4**		
	**BP**	**NP**	**SMD**	**BP**	**NP**	**SMD**	**BP**	**NP**	**SMD**	**BP**	**NP**	**SMD**	**BP**	**NP**	**SMD**
**# of episodes**	112345	126444		3915	69680		25728	38635		35313	15125		47375	2995	
**Age**															
Mean	73.0	73.0	0.0	72.3	72.9	-0.1	72.9	73.1	0.0	72.9	72.9	0.0	73.3	73.0	0.0
SD	8.2	8.4		8.7	8.5		8.4	8.3		8.4	8.5		8.0	8.5	
**Elixhauser score**															
Mean	4.3	4.3	0.0	4.8	4.1	0.1	4.5	4.5	0.0	4.3	4.6	0.0	4.1	4.4	0.0
SD	10.5	10.6		11.0	10.5		10.8	10.8		10.6	11.1		10.2	10.3	
**Black, %**	7.3	5.4	0.1	5.9	5.5	0.0	5.7	4.8	0.0	8.4	7.2	0.0	7.5	3.9	0.2
**Female, %**	63.9	62.6	0.0	60.1	62.6	-0.1	63.2	62.8	0.0	64.0	62.0	0.0	64.6	63.2	0.0
**Dual Eligible, %**	11.3	12.1	0.0	11.3	12.2	0.0	15.2	12.2	0.1	12.0	12.2	0.0	8.6	9.1	0.0
**Prior acute use, %**	14.6	16.5	-0.1	15.1	15.8	0.0	15.6	16.9	0.0	15.3	18.5	-0.1	13.4	17.4	-0.1
**Prior IRF use, %**	1.2	1.4	0.0	1.3	1.2	0.0	1.3	1.5	0.0	1.4	1.8	0.0	1.0	1.1	0.0
**Prior SNF use, %**	3.9	4.5	0.0	4.9	4.3	0.0	4.2	4.7	0.0	4.1	5.0	0.0	3.6	5.3	-0.1
**Coparticipant vs non-participant**	**Original Observed Data**			**IV Quartile 1**			**IV Quartile 2**			**IV Quartile 3**			**IV Quartile 4**		
	**CP**	**NP**	**SMD**	**CP**	**NP**	**SMD**	**CP**	**NP**	**SMD**	**CP**	**NP**	**SMD**	**CP**	**NP**	**SMD**
**# of episodes**	65411	126444		2448	69680		11681	38635		25606	15125		25674	2995	
**Age**															
Mean	73.0	73.0	0.0	72.3	72.9	-0.1	72.3	73.1	-0.1	73.0	72.9	0.0	73.2	73.0	0.0
SD	8.1	8.4		8.2	8.5		8.5	8.3		8.0	8.5		8.1	8.5	
**Elixhauser score**															
Mean	4.0	4.3	0.0	4.3	4.1	0.0	4.1	4.5	0.0	4.1	4.6	-0.1	3.8	4.4	-0.1
SD	10.3	10.6		10.9	10.5		10.3	10.8		10.4	11.1		10.1	10.3	
**Black, %**	5.5	5.4	0.0	3.0	5.5	-0.1	6.4	4.8	0.1	5.1	7.2	-0.1	5.6	3.9	0.1
**Female, %**	63.8	62.6	0.0	59.6	62.6	-0.1	62.4	62.8	0.0	63.2	62.0	0.0	65.5	63.2	0.0
**Dual Eligible, %**	9.1	12.1	-0.1	9.1	12.2	-0.1	10.7	12.2	0.0	8.5	12.2	-0.1	9.0	9.1	0.0
**Prior acute use, %**	14.6	16.5	-0.1	14.4	15.8	0.0	15.8	16.9	0.0	14.7	18.5	-0.1	13.9	17.4	-0.1

© 2019 Liao JM et al. *JAMA Network Open.*

**Prior IRF use, %**	1.3	1.4	0.0	1.5	1.2	0.0	1.3	1.5	0.0	1.3	1.8	0.0	1.2	1.1	0.0
**Prior SNF use, %**	4.0	4.5	0.0	4.3	4.3	0.0	4.0	4.7	0.0	4.1	5.0	0.0	3.9	5.3	-0.1

BP=bundled payment participant. CP=Co-participant. IRF=Inpatient Rehabilitation Facility. NP=Non-participant. SMD=Standardized mean difference. SNF=Skilled Nursing Facility.

© 2019 Liao JM et al. *JAMA Network Open.*

eTable 3. Patient and market characteristics by participation status (co-participants vs bundled payment participants) and study period, 2012-2016

	Co-participants			Bundled Payment Participants				
	Pre-bundled payment	Bundled payment	Difference	Pre-bundled payment	Bundled payment	Difference	Difference-in-Differences Estimate	Difference-in-Differences p-value
**Patient Characteristics[a]**								
**Age, mean year (SD)**	73.4 (1.9)	73.0 (1.7)	-0.4	73.4 (1.9)	73.0 (1.6)	-0.3	-0.03	0.92
**Black, % (SD)[b]**	5.9 (6.5)	6.0 (6.7)	0.1	9.2 (12.3)	9.0 (12.7)	-0.2	0.3	0.88
**Female, % (SD)**	65.5 (4.3)	64.6 (3.6)	-1.0	66.1 (6.0)	64.3 (4.1)	-1.9	0.9	0.25
**Dual-eligible, % (SD)[c]**	13.3 (9.7)	12.0 (9.3)	-1.4	16.7 (11.5)	15.1 (10.7)	-1.7	0.3	0.87
**Elixhauser comorbidity index, mean (SD)[d,e]**	5.1 (2.0)	4.4 (1.7)	-0.6	5.7 (3.0)	4.8 (2.0)	-0.9	0.3	0.53
**Prior acute care hospital use, % (SD)[e]**	17.6 (4.4)	15.6 (3.8)	-1.9	18.7 (7.7)	16.1 (4.5)	-2.6	0.7	0.48
**Prior IRF use, % (SD)[e]**	1.4 (1.4)	1.3 (1.1)	-0.2	1.6 (2.1)	1.5 (1.8)	-0.2	-0.02	0.94
**Prior SNF use, % (SD)[e]**	5.1 (3.2)	4.8 (2.6)	-0.3	5.4 (4.2)	4.8 (2.5)	-0.6	0.4	0.52
**Market Characteristics**								
**Quarterly LEJR volume, median (IQR)**	6.3 (4.9)	27.9 (13.3)	21.6	4.0 (4.2)	18.8 (11.2)	14.8	6.8	0.001
**Hospital beds, median (IQR)**	24.4 (11.4)	28.1 (11.7)	3.7	27.6 (12.7)	31.6 (13.3)	4.0	-0.3	0.92
**SNF beds, median (IQR)**	2,215.2 (1,727.7)	2,221.7 (1,700.9)	6.5	2,621.7 (1,771.3)	2,681.5 (1,824.3)	59.8	-53.3	0.90
**MA penetration, mean % (SD)**	815.0 (568.5)	779.9 (501.7)	-35.1	1,184.1 (896.1)	1,159.1 (886.1)	-24.9	-10.2	0.95
**ACO penetration, mean % (SD)**	6,243.3 (6,387.2)	6,277.1 (6,424.2)	33.9	5,189.4 (5,656.5)	5,243.9 (5,705.7)	54.5	-20.7	>0.99
**Hospital HHI, mean (SD)**	9,711.5 (8,247.8)	9,724.4 (8,269.7)	12.9	7,721.9 (7,392.3)	7,737.4 (7,429.8)	15.6	-2.6	>0.99
**SNF HHI, mean (SD)**	546.8 (415.7)	599.7 (462.8)	52.8	432.9 (374.5)	473.4 (416.7)	40.5	12.4	0.90
**PGP market, %**	0.0	61.7	61.7	0.0	55.3	55.3	6.3	0.43

This table describes patient characteristics in the pre-bundled payment and bundled payments periods for Co-participant, Bundled payment participant, and Non-participant hospitals. Pre-bundled payment period=January 2011 to September 2013. Bundled payment period=October 2013 to December 2016 (data presented were drawn from LEJR episodes occurring through September 2016 in order to allow for 90-day post-discharge period). IRF=Inpatient Rehabilitation Facility. SNF=Skilled Nursing Facility. LEJR=Lower Extremity Joint Replacement. MA=Medicare Advantage.

© 2019 Liao JM et al. *JAMA Network Open.*

ACO=Accountable Care Organization. HHI=Herfindahl-Hirschman Index. PGP=Physician Group Practice. Two-way ANOVA was used to test the statistical significance of the difference-in-differences estimates. [a]Characteristics for Co-participant and Bundled payment participant patients were drawn from a 100% Medicare claims sample. These descriptive statistics reflect a mean of hospital means, meaning that we calculated mean or percentage for patient characteristics at the hospital level first and then computed descriptive statistics by status and study period. [b]Race was broken out as black versus others because of existing disparities in access to LEJR among black patients specifically. [c]Dual eligible indicates eligibility for both the Medicare and Medicaid programs as an indicator of low socioeconomic status. [d]The Elixhauser comorbidity score is an index of severity with a range of −32 to +92 with increasing scores highly correlated with increased probability of in-hospital death. [e]Calculated using data from the year prior to LEJR hospitalization.

© 2019 Liao JM et al. *JAMA Network Open.*

eTable 4. Patient and market characteristics by participation status (co-participants vs non-participants) and study period, 2012-2016

	Co-participants			Non-participants				
	**Pre-bundled payment**	**Bundled payment**	**Difference**	**Pre-bundled payment**	**Bundled payment**	**Difference**	**Difference-in-Differences Estimate**	**Difference-in-Differences p-value**
**Patient Characteristics[a]**								
**Age, mean year (SD)**	73.4 (1.9)	73.0 (1.7)	-0.4	73.2 (3.1)	73.0 (2.7)	-0.2	-0.2	0.66
**Black, % (SD)[b]**	5.9 (6.5)	6.0 (6.7)	0.1	6.3 (11.9)	6.0 (10.5)	-0.3	0.4	0.81
**Female, % (SD)**	65.5 (4.3)	64.6 (3.6)	-1.0	64.8 (13.2)	63.2 (10.2)	-1.6	0.7	0.69
**Dual-eligible, % (SD)[c]**	13.3 (9.7)	12.0 (9.3)	-1.4	18.0 (16.4)	17.1 (14.1)	-0.9	-0.5	0.83
**Elixhauser comorbidity index, mean (SD)[d,e]**	5.1 (2.0)	4.4 (1.7)	-0.6	5.5 (3.7)	5.0 (3.3)	-0.4	-0.2	0.64
**Prior acute care hospital use, % (SD)[e]**	17.6 (4.4)	15.6 (3.8)	-1.9	20.4 (12.7)	18.5 (9.9)	-1.9	-0.02	0.99
**Prior IRF use, % (SD)[e]**	1.4 (1.4)	1.3 (1.1)	-0.2	1.7 (4.5)	1.5 (2.9)	-0.2	0.02	0.97
**Prior SNF use, % (SD)[e]**	5.1 (3.2)	4.8 (2.6)	-0.3	5.5 (7.2)	5.7 (6.7)	0.2	-0.5	0.63
**Market Characteristics**								
**Quarterly LEJR volume, median (IQR)**	6.3 (4.9)	27.9 (13.3)	21.6	3.7 (4.5)	16.6 (12.4)	12.9	8.8	<0.001
**Hospital beds, median (IQR)**	24.4 (11.4)	28.1 (11.7)	3.7	24.6 (13.2)	28.4 (13.6)	3.8	-0.1	0.96
**SNF beds, median (IQR)**	2,215.2 (1,727.7)	2,221.7 (1,700.9)	6.5	3,166.3 (2,022.7)	3,207.9 (2,042.3)	41.6	-35.1	0.93
**MA penetration, mean % (SD)**	815.0 (568.5)	779.9 (501.7)	-35.1	1,504.4 (1,164.1)	1,424.4 (1,085.6)	-80.0	44.9	0.83
**ACO penetration, mean % (SD)**	6,243.3 (6,387.2)	6,277.1 (6,424.2)	33.9	3,586.3 (4,119.7)	3,620.2 (4,161.4)	33.9	-0.1	>0.99
**Hospital HHI, mean (SD)**	9,711.5 (8,247.8)	9,724.4 (8,269.7)	12.9	5,655.2 (5,715.3)	5,651.8 (5,734.7)	-3.4	16.3	0.99
**SNF HHI, mean (SD)**	546.8 (415.7)	599.7 (462.8)	52.8	331.2 (304.1)	359.1 (336.9)	27.9	25.0	0.72
**PGP market, %**	0.0	61.7	61.7	0.0	47.0	47.0	14.7	0.04

This table describes patient characteristics in the pre-bundled payment and bundled payments periods for Co-participant, Bundled payment participant, and Non-participant hospitals. Pre-bundled payment period=January 2011 to September 2013. Bundled payment period=October 2013 to December 2016 (data presented were drawn from LEJR episodes occurring through September 2016 in order to allow for 90-day post-discharge period). IRF=Inpatient Rehabilitation Facility. SNF=Skilled Nursing Facility. LEJR=Lower Extremity Joint Replacement. MA=Medicare Advantage. ACO=Accountable Care Organization. HHI=Herfindahl-Hirschman Index. PGP=Physician Group Practice. Two-way ANOVA was used to test the

© 2019 Liao JM et al. *JAMA Network Open.*

statistical significance of the difference-in-differences estimates. [a]Characteristics for Non-participant patients were drawn from a 20% Medicare claims sample while characteristics for Co-participant patients were drawn from a 100% sample. These descriptive statistics reflect a mean of hospital means, meaning that we calculated mean or percentage for patient characteristics at the hospital level first and then computed descriptive statistics by status and study period. [b]Race was broken out as black versus others because of existing disparities in access to LEJR among black patients specifically. [c]Dual eligible indicates eligibility for both the Medicare and Medicaid programs as an indicator of low socioeconomic status. [d]The Elixhauser comorbidity score is an index of severity with a range of −32 to +92 with increasing scores highly correlated with increased probability of in-hospital death. [e]Calculated using data from the year prior to LEJR hospitalization.

© 2019 Liao JM et al. *JAMA Network Open.*

eTable 5. Patient and market characteristics by participation status (bundled payment participants vs non-participants) and study period, 2012-2016

	Bundled Payment Participants			Non-participants				
	Pre-bundled payment	Bundled payment	Difference	Pre-bundled payment	Bundled payment	Difference	Difference-in-Differences Estimate	Difference-in-Differences p-value
**Patient Characteristics[a]**								
**Age, mean year (SD)**	73.4 (1.9)	73.0 (1.6)	-0.3	73.2 (3.1)	73.0 (2.7)	-0.2	-0.2	0.61
**Black, % (SD)[b]**	9.2 (12.3)	9.0 (12.7)	-0.2	6.3 (11.9)	6.0 (10.5)	-0.3	0.1	0.94
**Female, % (SD)**	66.1 (6.0)	64.3 (4.1)	-1.9	64.8 (13.2)	63.2 (10.2)	-1.6	-0.3	0.81
**Dual-eligible, % (SD)[c]**	16.7 (11.5)	15.1 (10.7)	-1.7	18.0 (16.4)	17.1 (14.1)	-0.9	-0.8	0.63
**Elixhauser comorbidity index, mean (SD)[d,e]**	5.7 (3.0)	4.8 (2.0)	-0.9	5.5 (3.7)	5.0 (3.3)	-0.4	-0.5	0.18
**Prior acute care hospital use, % (SD)[e]**	18.7 (7.7)	16.1 (4.5)	-2.6	20.4 (12.7)	18.5 (9.9)	-1.9	-0.7	0.54
**Prior IRF use, % (SD)[e]**	1.6 (2.1)	1.5 (1.8)	-0.2	1.7 (4.5)	1.5 (2.9)	-0.2	0.0	0.91
**Prior SNF use, % (SD)[e]**	5.4 (4.2)	4.8 (2.5)	-0.6	5.5 (7.2)	5.7 (6.7)	0.2	-0.8	0.23
**Market Characteristics**								
**Quarterly LEJR volume, median (IQR)**	4.0 (4.2)	18.8 (11.2)	14.8	3.7 (4.5)	16.6 (12.4)	12.9	2.0	0.18
**Hospital beds, median (IQR)**	27.6 (12.7)	31.6 (13.3)	4.0	24.6 (13.2)	28.4 (13.6)	3.8	0.2	0.94
**SNF beds, median (IQR)**	2,621.7 (1,771.3)	2,681.5 (1,824.3)	59.8	3,166.3 (2,022.7)	3,207.9 (2042.3)	41.6	18.2	0.96
**MA penetration, mean % (SD)**	1,184.1 (896.1)	1,159.1 (886.1)	-24.9	1,504.4 (1,164.1)	1,424.4 (1,085.6)	-80.0	55.1	0.75
**ACO penetration, mean % (SD)**	5,189.4 (5,656.5)	5,243.9 (5,705.7)	54.5	3,586.3 (4,119.7)	3,620.2 (4161.4)	33.9	20.6	0.98
**Hospital HHI, mean (SD)**	7,721.9 (7,392.3)	7,737.4 (7,429.8)	15.6	5,655.2 (5,715.3)	5,651.8 (5,734.7)	-3.4	18.9	0.99
**SNF HHI, mean (SD)**	432.9 (374.5)	473.4 (416.7)	40.5	331.2 (304.1)	359.1 (336.9)	27.9	12.6	0.82
**PGP market, %**	0.0	55.3	55.3	0	47.0	47.0	8.4	0.15

This table describes patient characteristics in the pre-bundled payment and bundled payments periods for Co-participant, Bundled payment participant, and Non-participant hospitals. Pre-bundled payment period=January 2011 to September 2013. Bundled payment period=October 2013 to December 2016 (data presented were drawn from LEJR episodes occurring through September 2016 in order to allow for 90-day post-discharge

© 2019 Liao JM et al. *JAMA Network Open.*

period). IRF=Inpatient Rehabilitation Facility. SNF=Skilled Nursing Facility. LEJR=Lower Extremity Joint Replacement. MA=Medicare Advantage. ACO=Accountable Care Organization. HHI=Herfindahl-Hirschman Index. PGP=Physician Group Practice. Two-way ANOVA was used to test the statistical significance of the difference-in-difference estimates. [a]Characteristics for Non-participant patients were drawn from a 20% Medicare claims sample while characteristics for Bundled payment participant patients were drawn from a 100% sample. These descriptive statistics reflect a mean of hospital means, meaning that we calculated mean or percentage for patient characteristics at the hospital level first and then computed descriptive statistics by status and study period. [b]Race was broken out as black versus others because of existing disparities in access to LEJR among black patients specifically. [c]Dual eligible indicates eligibility for both the Medicare and Medicaid programs as an indicator of low socioeconomic status. [d]The Elixhauser comorbidity score is an index of severity with a range of −32 to +92 with increasing scores highly correlated with increased probability of in-hospital death. [e]Calculated using data from the year prior to LEJR hospitalization.

© 2019 Liao JM et al. *JAMA Network Open.*

eTable 6. Unadjusted changes in clinical outcomes and spending associated with participation status (co-participants vs bundled payment participants), 2012-2016

	Co-participants				Bundled Payment Participants						
	Pre-bundled payment	Bundled payment	Difference, %	p-value[a]	Pre-bundled payment	Bundled payment	Difference, %	p-value[a]	Difference in change[b]	Differential change, %	p-value[c]
**Primary Clinical Outcomes and Spending**											
**Mortality rate, %**	1.8	1.6	-12.3	0.007	2.0	1.7	-14.0	<0.001	0.1	-20.1	0.60
**Unplanned 90-day readmission rate, %**	8.8	8.0	-8.8	<0.001	11.1	8.7	-21.2	<0.001	1.6	-66.9	<0.001
**ED visit rate, %[d]**	13.5	13.1	-2.5	0.13	13.4	13.6	1.0	0.42	-0.5	-344.0	0.09
**LEJR-specific complication rate, %[e]**	3.7	3.6	-2.1	0.51	3.7	3.6	-3.3	0.19	0.0	-34.8	0.78
**Episode spending, $ (SD)**	23,142 (14,257)	21,657 (12,720)	-6.4	<0.001	24,298 (14,167)	22,650 (14,009)	-6.8	<0.001	163.4	-9.9	0.14
**Secondary Clinical Outcomes**											
**Index hospitalization LOS, mean days (SD)**	3.9 (5.0)	3.3 (3.2)	-15.2	<0.001	4.2 (4.1)	3.8 (4.6)	-8.0	<0.001	-0.3	75.2	<0.001
**Post-discharge follow-up, %**	28.6	28.1	-1.7	0.09	25.9	24.5	-5.4	<0.001	0.9	-64.6	0.01
**Discharge with HHA, %**	63.8	63.0	-1.2	0.01	71.0	71.1	0.2	0.57	-0.9	-714.0	0.02
**Discharge to institutional PAC provider, %**	32.0	24.1	-24.7	<0.001	36.3	27.8	-23.5	<0.001	0.6	-7.5	0.08
**30-day readmission rate, %**	5.6	4.8	-13.7	<0.001	7.6	5.5	-27.6	<0.001	1.3	-63.3	<0.001
**60-day readmission rate, %**	7.6	6.7	-11.4	<0.001	9.6	7.4	-22.8	<0.001	1.3	-60.6	<0.001

Pre-bundled payment=January 2012 to September 2013; Bundled payment=October 2013 to December 2016. All spending estimates were standardized and adjusted for inflation and transformed into 2016 dollars. LEJR=Lower Extremity Joint Replacement. LOS=length of stay. HHA=Home Health Agency. Institutional PAC provider=skilled nursing facility or inpatient rehabilitation facility. [a]Obtained from Wilcoxon rank sum tests for continuous outcomes and Chi squared tests for categorical outcomes. [b]Calculated by subtracting the difference between pre-bundled payment and bundled payment periods among patients in one participation group from the difference between pre-bundled payment and bundled payment periods among the comparison participation group. [c]Obtained from two-way ANOVA, with p-value reflecting statistical significance of the interaction term measuring differential change. [d]Emergency Department (ED) visits without hospitalization. [e]Defined by Hospital Compare.

© 2019 Liao JM et al. *JAMA Network Open.*

eTable 7. Unadjusted changes in clinical outcomes and spending associated with participation status (co-participants vs non- participants), 2012-2016

	Co-participants				Non-participants						
	**Pre-bundled payment**	**Bundled payment**	**Difference, %**	**p-value[a]**	**Pre-bundled payment**	**Bundled payment**	**Difference, %**	**p-value[a]**	**Difference in change[b]**	**Differential change, %**	**p-value[c]**
**Primary Clinical Outcomes and Spending**											
**Mortality rate, %**	1.8	1.6	-12.3	0.01	2.1	2.0	-5.5	0.07	-0.1	89.1	0.34
**Unplanned 90-day readmission rate, %**	8.8	8.0	-8.8	<0.001	9.6	8.9	-7.1	<0.001	-0.1	14.2	0.67
**ED visit rate, %[d]**	13.5	13.1	-2.5	0.13	6.7	6.2	-6.6	<0.001	0.1	-24.4	0.63
**LEJR-specific complication rate, %[e]**	3.7	3.6	-2.1	0.51	4.0	4.2	3.4	0.13	-0.2	-158.0	0.16
**Episode spending, $ (SD)**	23,142 (14,257)	21,657 (12,720)	-6.4	<0.001	23,145 (14,140)	22,413 (13,883)	-3.2	<0.001	-752.6	102.9	<0.001
**Secondary Clinical Outcomes**											
**Index hospitalization LOS, mean days (SD)**	3.9 (5.0)	3.3 (3.2)	-15.2	<0.001	3.9 (4.6)	4.0 (4.6)	2.4	<0.001	-0.7	-723.0	<0.001
**Post-discharge follow-up, %**	28.6	28.1	-1.7	0.09	31.2	31.6	1.2	0.07	-0.9	-230.0	0.02
**Discharge with HHA, %**	63.8	63.0	-1.2	0.01	60.1	58.1	-3.4	<0.001	1.2	-61.6	0.001
**Discharge to institutional PAC provider, %**	32.0	24.1	-24.7	<0.001	31.8	27.1	-14.6	<0.001	-3.2	69.6	<0.001
**30-day readmission rate, %**	5.6	4.8	-13.7	<0.001	5.9	5.4	-8.1	<0.001	-0.3	62.0	0.10
**60-day readmission rate, %**	7.6	6.7	-11.4	<0.001	8.1	7.4	-7.7	<0.001	-0.2	38.7	0.24

Pre-bundled payment=January 2012 to September 2013; Bundled payment=October 2013 to December 2016. All spending estimates were standardized and adjusted for inflation and transformed into 2016 dollars. LEJR=Lower Extremity Joint Replacement. LOS=length of stay. HHA=Home Health Agency. Institutional PAC provider=skilled nursing facility or inpatient rehabilitation facility. [a]Obtained from Wilcoxon rank sum tests for continuous outcomes and Chi squared tests for categorical outcomes. [b]Calculated by subtracting the difference between pre-bundled payment and bundled payment periods among patients in one participation group from the difference between pre-bundled payment and bundled payment periods among the comparison participation group. [c]Obtained from two-way ANOVA, with p-value reflecting statistical significance of the interaction term measuring differential change. [d]Emergency Department (ED) visits without hospitalization. [e]Defined by Hospital Compare.

© 2019 Liao JM et al. *JAMA Network Open.*

eTable 8. Unadjusted changes in clinical outcomes and spending associated with participation status (bundled payment participants vs non-participants), 2012-2016

	Bundled Payment Participants				Non-participants						
	Pre-bundled payment	Bundled payment	Difference, %	P-value[a]	Pre-bundled payment	Bundled payment	Difference, %	P-value[a]	Difference in change[b]	Differential change, %	P-value[c]
**Primary Clinical Outcomes and Spending**											
**Mortality rate, %**	2.0	1.7	-14.0	<0.001	2.1	2.0	-5.5	0.07	-0.2	136.6	0.09
**Unplanned 90-day readmission rate, %**	11.1	8.7	-21.2	<0.001	9.6	8.9	-7.1	<0.001	-1.7	245.3	<0.001
**ED visit rate, %[d]**	13.4	13.6	1.0	0.42	6.7	6.2	-6.6	<0.001	0.6	-131.0	0.004
**LEJR-specific complication rate, %[e]**	3.7	3.6	-3.3	0.19	4.0	4.2	3.4	0.13	-0.3	-189.0	0.05
**Episode spending, $ (SD)**	24,298 (14,167)	22,650 (14,009)	-6.8	<0.001	23,145 (14,140)	22,413 (13,883)	-3.2	<0.001	-916.0	125.2	<0.001
**Secondary Clinical Outcomes**											
**Index hospitalization LOS, mean days (SD)**	4.2 (4.1)	3.8 (4.6)	-8.0	<0.001	3.9 (4.6)	4.0 (4.6)	2.4	<0.001	-0.4	-456.0	<0.001
**Post-discharge follow-up, %**	25.9	24.5	-5.4	<0.001	31.2	31.6	1.2	0.07	-1.8	-467.0	<0.001
**Discharge with HHA, %**	71.0	71.1	0.2	0.57	60.1	58.1	-3.4	<0.001	2.1	-106.0	<0.001
**Discharge to institutional PAC provider, %**	36.3	27.8	-23.5	<0.001	31.8	27.1	-14.6	<0.001	-3.9	83.4	<0.001
**30-day readmission rate, %**	7.6	5.5	-27.6	<0.001	5.9	5.4	-8.1	<0.001	-1.6	341.7	<0.001
**60-day readmission rate, %**	9.6	7.4	-22.8	<0.001	8.1	7.4	-7.7	<0.001	-1.6	252.0	<0.001

Pre-bundled payment=January 2012 to September 2013; Bundled payment=October 2013 to December 2016. All spending estimates were standardized and adjusted for inflation and transformed into 2016 dollars. LEJR=Lower Extremity Joint Replacement. LOS=length of stay. HHA=Home Health Agency. Institutional PAC provider=skilled nursing facility or inpatient rehabilitation facility. [a]Obtained from Wilcoxon rank sum tests for continuous outcomes and Chi squared tests for categorical outcomes. [b]Calculated by subtracting the difference between pre-bundled

© 2019 Liao JM et al. *JAMA Network Open.*

payment and bundled payment periods among patients in one participation group from the difference between pre-bundled payment and bundled payment periods among the comparison participation group. [c]Obtained from two-way ANOVA, with p-value reflecting statistical significance of the interaction term measuring differential change. [d]Emergency Department (ED) visits without hospitalization. [e]Defined by Hospital Compare.

© 2019 Liao JM et al. *JAMA Network Open.*

eTable 9. Sensitivity analyses without use of an instrumental variable

	Bundled Payment Participants vs. Non-participants		Co-participants vs. Non-participants		Co-participants vs. Bundled Payment Participants	
	**Difference-in-Differences Estimate**	**p-value**	**Difference-in-Differences Estimate**	**p-value**	**Difference-in-Differences Estimate**	**p-value**
**Primary Clinical Outcomes and Spending**						
**Mortality rate**	0.04 (-0.2 to 0.1)	0.57	-0.2 (-0.4 to 0.0)	0.12	-0.1 (-0.3 to 0.1)	0.26
**Unplanned 90-day readmission rate**	-0.7 (-1.7 to 0.3)	0.16	0.4 (-0.4 to 1.1)	0.33	1.1 (-0.4 to 2.5)	0.15
**ED visit rate[a]**	0.7 (0.3 to 1.1)	0.001	0.1 (-0.6 to 0.9)	0.77	-0.6 (-1.3 to 0.2)	0.15
**LEJR-specific complication rate[b]**	0.1 (-0.2 to 0.3)	0.68	-0.2 (-0.5 to 0.2)	0.37	-0.2 (-0.6 to 0.2)	0.28
**Episode spending**	-3.1 (-4.2 to -2.0)	<0.001	-2.6 (-4.2 to -0.9)	0.003	0.5 (-1.5 to 2.7)	0.61

This table shows results from difference-in-differences models evaluating the association between participation status and differential changes in clinical outcomes and spending without the use of the instrumental variable. Negative estimates indicate reductions in rates (i.e., improved clinical outcomes or reduced spending). LEJR=Lower Extremity Joint Replacement. [a]Emergency Department (ED) visits without hospitalization. [b]Defined by Hospital Compare.

© 2019 Liao JM et al. *JAMA Network Open.*

eTable 10. Sensitivity analyses excluding January-September 2013

	Bundled Payment Participants vs. Non-participants		Co-participants vs. Non-participants		Co-participants vs. Bundled Payment Participants	
	Difference-in-Differences Estimate	p-value	Difference-in-Differences Estimate	p-value	Difference-in-Differences Estimate	p-value
**Primary Clinical Outcomes and Spending**						
**Mortality rate**	-0.02 (-0.27 to 0.23)	0.88	-0.12 (-0.50 to 0.25)	0.52	-0.1 (-0.47 to 0.27)	0.59
**Unplanned 90-day readmission rate**	-1.26 (-1.80 to -0.72)	<0.001	0.08 (-0.75 to 0.91)	0.85	1.34 (0.51 to 2.18)	<0.001
**ED visit rate[a]**	0.43 (-0.17 to 1.03)	0.16	-0.35 (-1.25 to 0.54)	0.44	-0.78 (-1.69 to 0.13)	0.09
**Episode LEJR complication rate[b]**	0.00 (-0.35 to 0.35)	>0.99	-0.12 (-0.64 to 0.41)	0.66	-0.12 (-0.67 to 0.44)	0.68
**Episode spending**	-3.26 (-4.01 to -2.51)	<0.001	-2.75 (-3.95 to -1.54)	<0.001	0.52 (-0.75 to 1.82)	0.42

This table shows results from difference-in-differences models evaluating the association between participation status and differential changes in clinical outcomes and spending, excluding the period between January and September 2013. Negative estimates indicate reductions in rates (i.e., improved clinical outcomes or reduced spending). LEJR=Lower Extremity Joint Replacement. [a]Emergency Department (ED) visits without hospitalization. [b]Defined by Hospital Compare.

© 2019 Liao JM et al. *JAMA Network Open.*

eTable 11. Sensitivity analyses using an intention-to-treatment approach to assigning hospital BPCI status

	**Bundled Payment Participants vs. Non-participants**		**Co-participants vs. Non-participants**		**Co-participants vs. Bundled Payment Participants**	
	**Difference-in-Differences Estimate**	**p-value**	**Difference-in-Differences Estimate**	**p-value**	**Difference-in-Differences Estimate**	**p-value**
**Primary Clinical Outcomes and Spending**						
**Mortality rate**	-0.03 (-0.25 to 0.19)	0.78	-0.16 (-0.49 to 0.17)	0.34	-0.13 (-0.46 to 0.20)	0.44
**Unplanned 90-day readmission rate**	-1.32 (-1.83 to -0.81)	<.001	0.13 (-0.57 to 0.83)	0.71	1.45 (0.73 to 2.18)	<.001
**ED visit rate[a]**	0.34 (-0.22 to 0.90)	0.23	-0.15 (-0.96 to 0.65)	0.71	-0.50 (-1.33 to 0.34)	0.24
**Episode LEJR complication rate[b]**	0.02 (-0.30 to 0.34)	0.90	-0.11 (-0.57 to 0.36)	0.65	-0.13 (-0.60 to 0.35)	0.61
**Episode spending**	-3.32 (-4.04 to -2.59)	<.001	-2.69 (-3.80 to -1.57)	<.001	0.65 (-0.45 to 1.76(	0.25

This table shows results from difference-in-differences models evaluating the association between participation status and differential changes in clinical outcomes and spending, using an intention-to-treat approach to assigning hospital BPCI participation. Negative estimates indicate reductions in rates (i.e., improved clinical outcomes or reduced spending). LEJR=Lower Extremity Joint Replacement. [a]Emergency Department (ED) visits without hospitalization. [b]Defined by Hospital Compare.

© 2019 Liao JM et al. *JAMA Network Open.*

eMethods 1. Instrumental variable approach

We introduce a novel instrumental variable (IV) to mitigate confounding from selection of beneficiaries for hospitalization based on unobservable characteristics. This IV is an adaptation of instrumental variables from outside of healthcare.[1,2] Our approach uses historical hospital referral patterns before the beginning of the BPCI program to identify the subset of patients hospitalized for LEJR at bundled payment participant hospitals in the bundled payment period, but who were hospitalized at these hospitals because of historical referral patterns. Specifically, we used patient characteristics (e.g., sociodemographics, clinical conditions, ZIP code of residence, primary care physician) from 2011 in the pre-bundled payment period to predict the probability that a given patient would be hospitalized for LEJR at a hospital that later participates in BPCI (bundled payment participant hospital).

Importantly, we could not use other standard IVs used in health care services research such as the distance to hospital. This is because many hospital systems in bundled payment could have exerted substantial effort to attract preferred (i.e., lower risk or healthier) types of patients, many of whom were at greater distance from the hospital. These efforts could include buying hospitals in outlying suburban areas, buying physician practices, or contracting with surgeons in areas with a greater density of preferred patients.

Because historical hospital referral patterns for elective conditions such as LEJR are not correlated with changes in patient selection after hospital participation in BPCI (conditional on variables we can observe in our data such as patient demographics), they serve as a reasonable IV for our analysis. In tests of the instrumental variable with respect to observed treatment at a bundled payment participant hospital, we found a strong association (F-measure of 11,835) between predicted and observed BPCI exposure, controlling for time-varying hospital, market, and patient characteristics and including hospital, market, and time fixed-effects. This confirmed that we had a strong instrument that was uncorrelated with the confounder of unobservable patient selection, but highly predictive of our treatment, i.e., admission to a bundled payment participant hospital (in

© 2019 Liao JM et al. *JAMA Network Open.*

the bundled payment period). The instrument was constructed using 2011 data and the formula $BPCI_{ever,h} = Zip_{pt} + Cov_{pt} + \varepsilon$, where $BPCI_{ever,h}$ is an indicator describing whether a Medicare beneficiary was admitted for LEJR at a hospital that later joins BPCI (at any point), Zip_{pt} is the beneficiary's ZIP code of residence, Cov_{pt} is a vector of characteristics of the beneficiary (including demographics and clinical comorbidities) and ε is the error term.

In the first-stage regression, we used pre-bundled payment period hospital referral patterns in 2011 to generate the predicted probability of hospitalization for LEJR at an eventual bundled payment participant hospital ($\bar{\pi}_{pt}$), and used that probability as an instrument for actual hospitalization for LEJR at a bundled payment participant hospital. The first stage is $BPCI\_observed_{pt,h,t} = \bar{\pi}_{pt} + HRR_{FE} + Time_{FE} + Cov_{pt} + Cov_{HRR} + \varepsilon$, where the observed bundled payment participation status for a beneficiary receiving LEJR (whether the hospital the beneficiary was admitted to participated in BPCI in that market-quarter) was regressed on the IV ($\bar{\pi}_{pt}$, the predicted probability for that beneficiary of going to a bundled payment participant hospital based on historical patterns), market (based on patient ZIP code of residence) and time fixed effects, patient characteristic covariates, market time-varying covariates, and an error term. In the second stage regression, we then used hospitalization to a bundled payment participant hospital as an instrument for observed "treatment" at a bundled payment or non-participant hospital in the period after BPCI began, relating treatment to spending and quality outcomes. $y_{pt,h,t} = \alpha + \beta * BPCI\_observed_{pt,h,t} + \gamma * Hosp_{FE} + HRR_{FE} + Time_{FE} + \delta * Cov_{pt} + \theta * Cov_{HRR} + \varepsilon$, where the coefficient of interest is β and captures the average effect of BPCI on outcome y. While we show these equations separately, this was estimated simultaneously using 2 stage least squares (2SLS) and not in 2 steps. This instrumental variable approach allowed us to measure the effect of BPCI among patients who received LEJR at a bundled payment participant hospital regardless of BPCI's existence. We also conducted a Hausman test evaluating for endogeneity, finding p<0.001 and therefore rejecting the null hypothesis of equivalence (i.e., no endogeneity from unobserved confounding). This result provided additional

© 2019 Liao JM et al. *JAMA Network Open.*

rationale for the need for this IV to mitigate confounding from unobserved selection. We used bootstrapped standard errors to account for the fact that the IV is an estimated quantity.[3]

© 2019 Liao JM et al. *JAMA Network Open.*

eMethods 2. Tests of parallel trends between hospital groups for primary clinical outcomes and spending variables

Tests of parallel trends were evaluated using generalized linear regression models for primary clinical and spending outcomes as the dependent variable, and independent variables of categorical time (quarter) fixed effects, time varying BPCI Model 2 participation indicator variable, their interaction, as well as an ACO participation indicator variable. The table demonstrates that overall, the time fixed effects-BPCI participation interaction term coefficients are not statistically significant, indicating no divergent trends in the pre-period for evaluated outcomes.

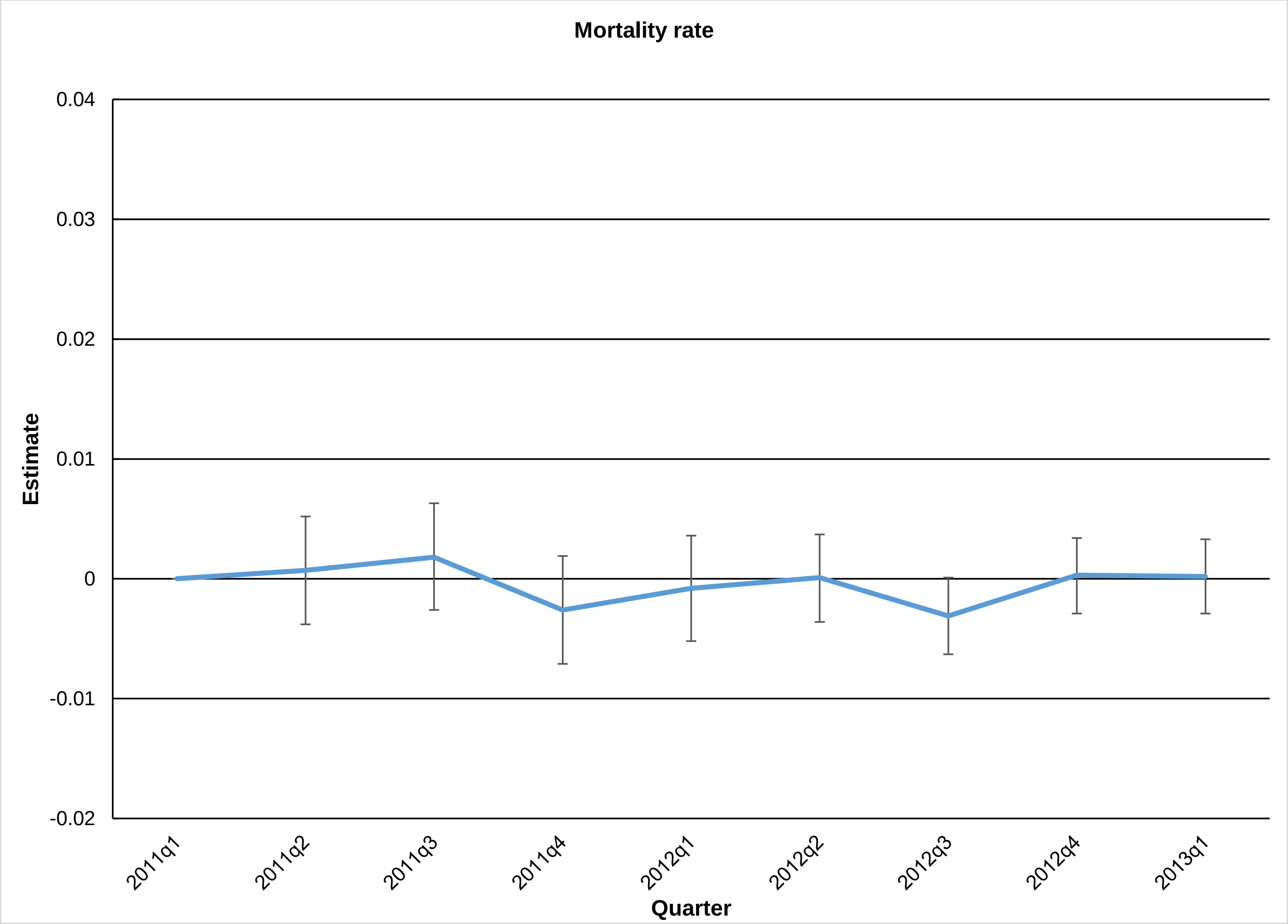

© 2019 Liao JM et al. *JAMA Network Open.*

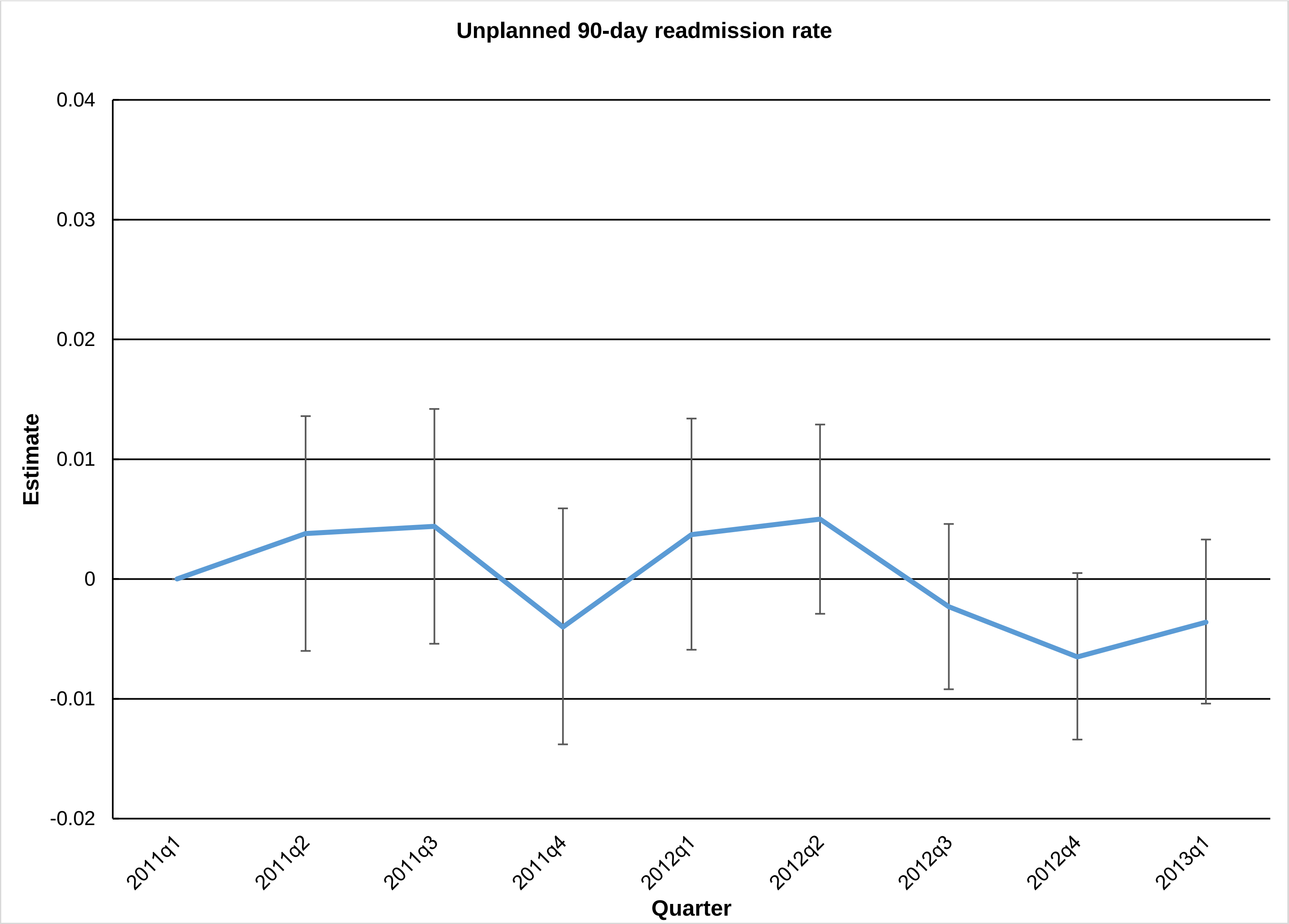

© 2019 Liao JM et al. *JAMA Network Open.*

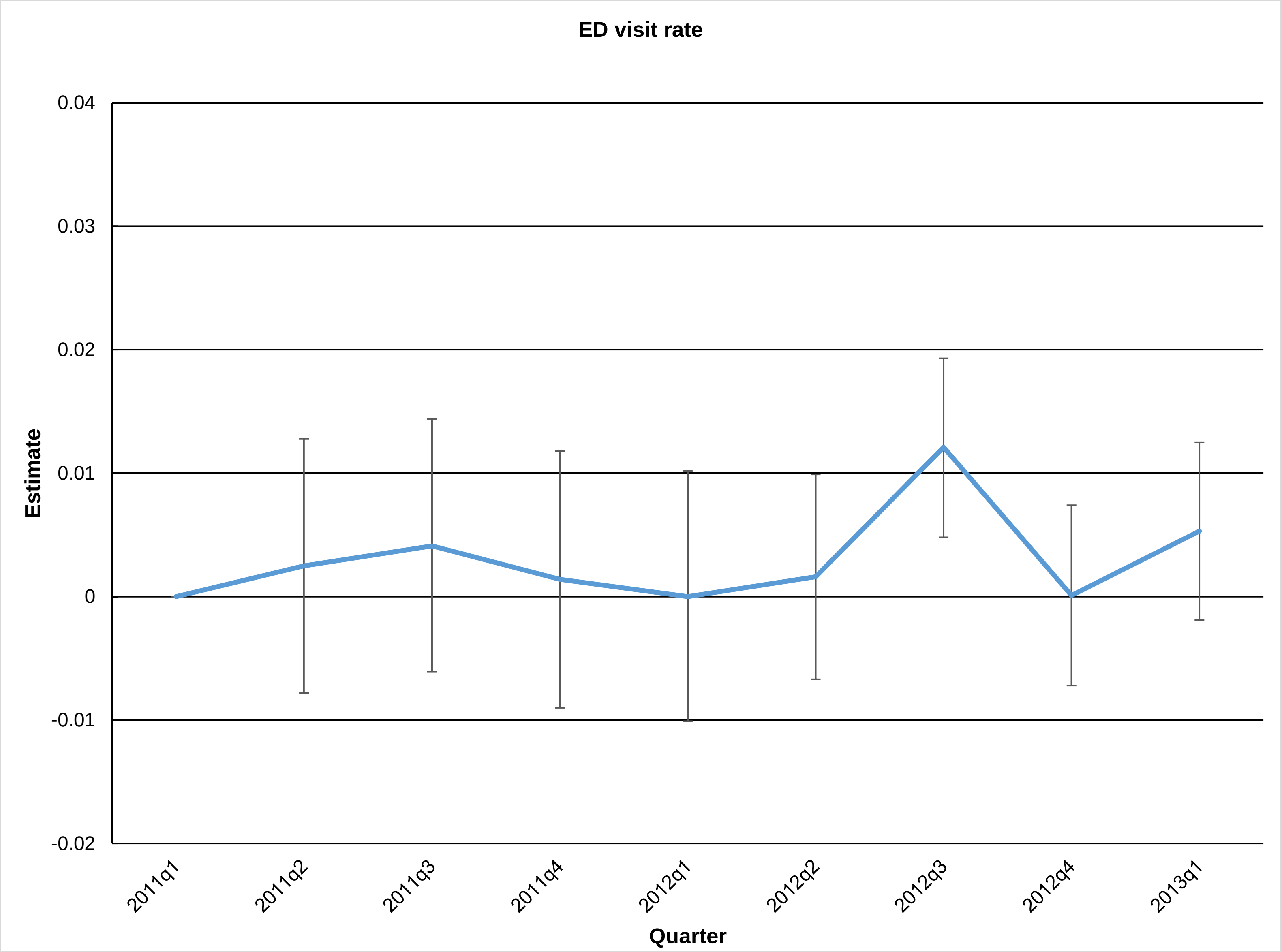

© 2019 Liao JM et al. *JAMA Network Open.*

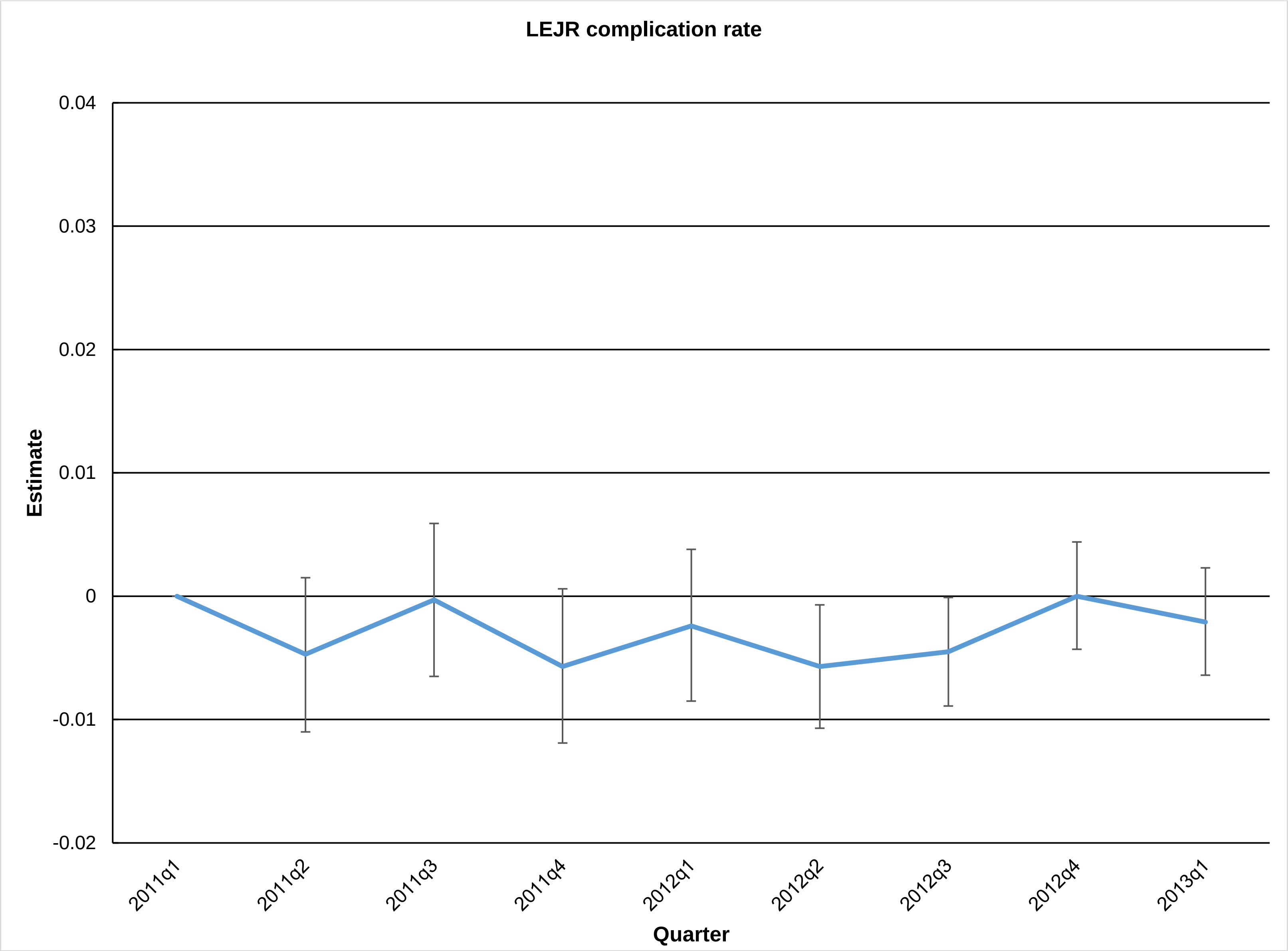

© 2019 Liao JM et al. *JAMA Network Open.*

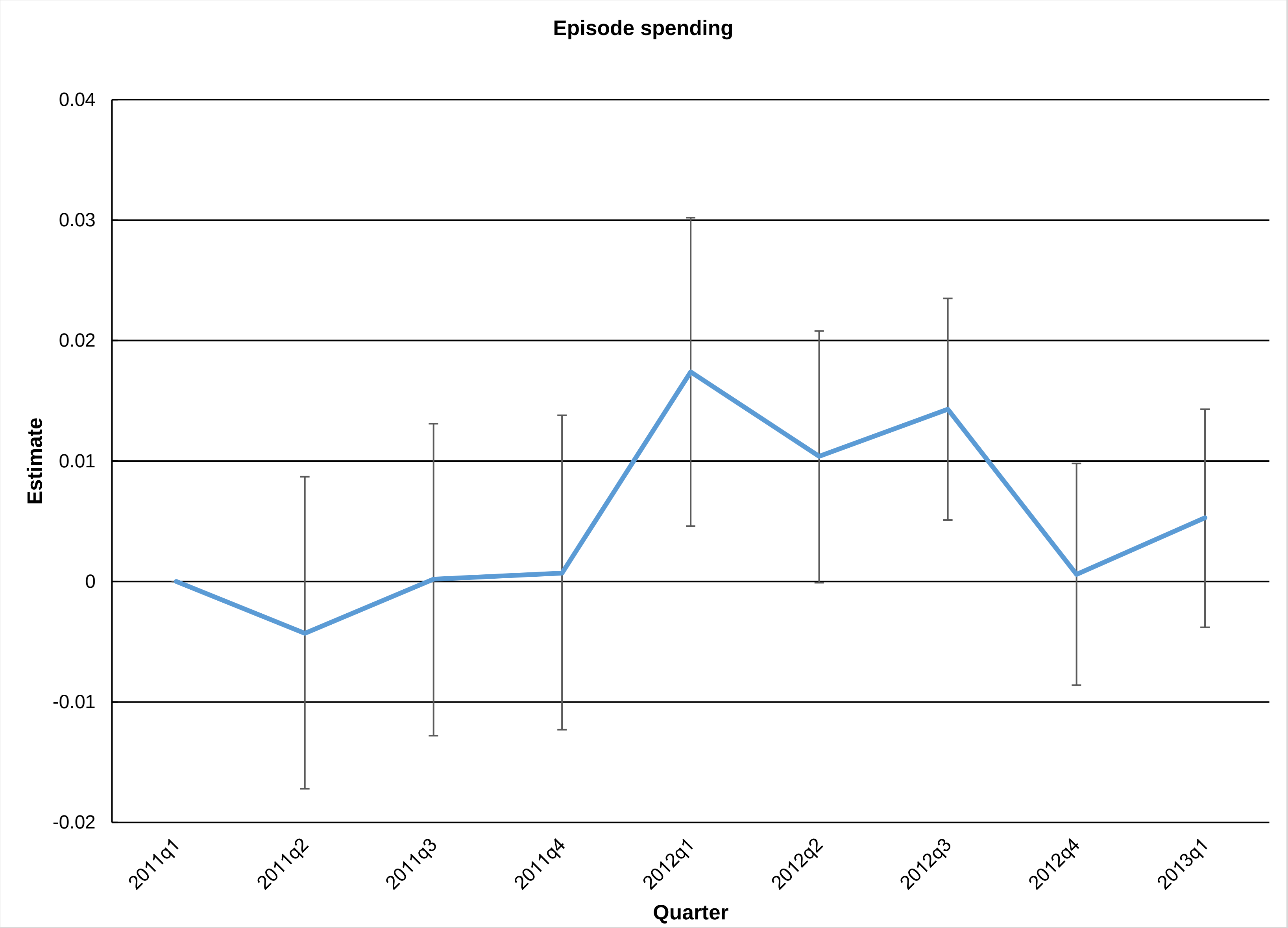

© 2019 Liao JM et al. *JAMA Network Open.*

eReferences

1. Imbens GW, Angrist JD. Identification and estimation of local average treatment effects. *Econometrica*. 1994;62(2):467-475.
2. Angrist JD, Krueger AB. Instrumental variables and the search for identification: from supply and demand to natural experiments. *J Econ Perspect.* 2001;15(4):69-85.
3. Angrist J, Pischke J. *Mostly Harmless Econometrics: An Empiricist's Companion.* Princeton, NJ: Princeton University Press; 2008.

© 2019 Liao JM et al. *JAMA Network Open.*
